# Selection of suitable reference genes for qRT-PCR normalization during leaf development and hormonal stimuli in tea plant (*Camellia sinensis*)

**DOI:** 10.1038/srep19748

**Published:** 2016-01-27

**Authors:** Zhi-Jun Wu, Chang Tian, Qian Jiang, Xing-Hui Li, Jing Zhuang

**Affiliations:** 1 Tea Science Research Institute, College of Horticulture, Nanjing Agricultural University, Nanjing, 210095, China; 2 State Key Laboratory of Crop Genetics and Genetics and Germplasm Enhancement, College of Horticulture, Nanjing Agricultural University, Nanjing, 210095, China

## Abstract

Tea plant (*Camellia sinensis*) leaf is an important non-alcoholic beverage resource. The application of quantitative real time polymerase chain reaction (qRT-PCR) has a profound significance for the gene expression studies of tea plant, especially when applied to tea leaf development and metabolism. In this study, nine candidate reference genes (i.e., *CsACT7*, *CsEF-1α*, *CseIF-4α, CsGAPDH*, *CsPP2A*, *CsSAND*, *CsTBP*, *CsTIP41*, and *CsTUB*) of *C. sinensis* were cloned. The quantitative expression data of these genes were investigated in five tea leaf developmental stages (i.e., 1st, 2nd, 3rd, 4th, and older leaves) and normal growth tea leaves subjected to five hormonal stimuli (i.e., ABA, GA, IAA, MeJA, and SA), and gene expression stability was calculated using three common statistical algorithms, namely, geNorm, NormFinder, and Bestkeeper. Results indicated that *CsTBP* and *CsTIP41* were the most stable genes in tea leaf development and *CsTBP* was the best gene under hormonal stimuli; by contrast, *CsGAPDH* and *CsTUB* genes showed the least stability. The gene expression profile of *CsNAM* gene was analyzed to confirm the validity of the reference genes in this study. Our data provide basis for the selection of reference genes for future biological research in the leaf development and hormonal stimuli of *C. sinensis*.

Quantifying gene expression is a common technique in molecular biology studies. Quantitative real time polymerase chain reaction (qRT-PCR) has become the most prevalent method applied to quantify assays of gene expression^1^,^2^. The two methods of presenting quantitative gene expression include the absolute and relative quantification methods. Absolute quantification provides an exact copy number of genes by transforming quantification cycle in a standard curve^3^. Relative quantification presents real-time PCR data of target genes relying on internal control genes as reference^4^. As knowledge of the copy number of gene is often unnecessary and most researchers focus on the discrepant analysis of gene expression, relative quantification has become a more common and powerful tool for gene expression assays^5^. Considering its many benefits, however, relative quantification is disadvantaged by the fact that at least one stable internal reference gene must be employed during analysis. Selection of a reliable reference gene under certain conditions is the key to quantitative accuracy.

Many genes are involved in the maintenance of basic cellular functions, such as the cell structure and primary metabolism. Some of these genes, such as the actin7 gene (*ACT7*), elongation factor-1α gene (*EF-1α*), eukaryotic translation initiation factor 4α-1 gene (*eIF-4α*), glyceraldehyde-3-phosphate dehydrogenase gene (*GAPDH*), protein phosphatase 2A gene (*PP2A*), SAND family protein gene (*SAND*), TATA-box binding protein gene (*TBP*), Tap42-interacting protein of 41 kDa gene (*TIP41*), and tubulin beta gene (*TUB*), have been identified as reliable reference genes in plants^6^,^7^. A main assessment criterion for choosing suitable reference genes under certain conditions is that the gene should be stably expressed under the desired test conditions. Given that reference genes do not always show perfectly stable expression in response to a variety of conditions or cross species, reassessment of reference genes under certain conditions is essential to ensure the accuracy of the calculation results in gene expression studies^8^,^9^.

Tea plant (*Camellia sinensi*s (L.) O. Kuntze) is an important leaf-type woody crop used for the production of non-alcoholic beverages worldwide^10^,^11^. Tender tea leaves are rich in beneficial metabolites, including tea polyphenols, theanine, and polysaccharides, which exert positive effects on the prevention of cancer, cardiovascular, and neurodegenerative diseases^12^,^13^,^14^,^15^,^16^. The concentration of these active substances is usually affected by different leaf development stages and seasonal climates^17^,^18^,^19^. Therefore, tender tea leaves are picked as ideal beverage-processing materials within a suitable season, such as spring and summer. Tea leaves are frequently used as experimental materials in molecular biology studies, including research on the tea leaf transcriptome, metabolomic, and development^20^,^21^,^22^. However, no systematic analysis of reference gene selection for normalization in various tea leaf development stages and hormonal treatments is yet available.

In this study, nine common reference genes (*CsACT7*, *CsEF-1α*, *CseIF-4α, CsGAPDH*, *CsPP2A*, *CsSAND*, *CsTBP*, *CsTIP41*, and *CsTUB*) with high homology to *Arabidopsis* were cloned and identified in *C. sinensis*. These nine genes were selected to assess the stability of gene expression in five different developmental stages (1st, 2nd, 3rd, 4th, and older leaves) and five different hormonal stimuli (ABA, GA, IAA, MeJA, and SA) in tea plant. Three different statistical algorithms (geNorm^23^, NormFinder^24^, and BestKeeper^25^) were used to calculate the variability of the expression of the candidate genes and obtain the most suitable reference genes. This study provides a basis for the selection of reference genes and useful guidelines for future gene expression studies of *C. sinensis*.

## Results

### Cloning and quality control of candidate reference genes

**Figure 1:**
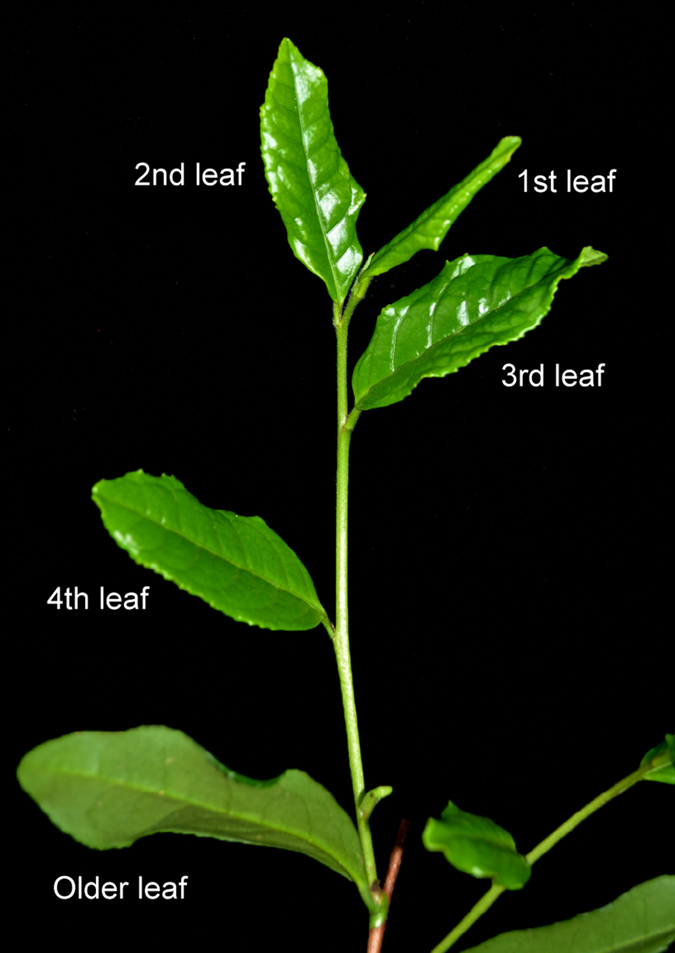
Photograph of the leaf tissue of the tea plant cultivar ‘Longjing43’. Relative position of leaves from a two-years-old cutting seedling of *C. sinensis* cv. ‘Longjing43’.

Based on homology analysis with *Arabidopsis*, the full-length sequences of nine candidate reference genes (*CsACT7*, *CsEF-1α*, *CseIF-4α, CsGAPDH*, *CsPP2A*, *CsSAND*, *CsTBP*, *CsTIP41*, and *CsTUB*) were identified from the *C. sinensis* transcriptome^20^. These nine genes were cloned from *C. sinensis* cv. ‘Longjing43’ (Fig. 1 and Supplementary Fig. S1). The sequence information of these genes is shown in Supplementary Figs S2–S10. The minimal identity of the encoded sequences of all of the genes at the amino acid level to the target homologs exceeded 64%, and five cloned genes showed high identities exceeding 90% (Supplementary Table S1). Specific primer pairs were designed, and confirmed on the basis of the amplification specificity and efficiency results of the candidate reference genes: all primers were amplified with a single PCR product of the expected size by 1.2% agarose gel electrophoresis (Supplementary Fig. S11); single-peak melting curves were obtained in all qPCR amplifications; the amplification efficiency (E) of all reactions ranged from 95.5% to 107.5%; and the correlation coefficients (R^2^) of the standard curve varied from 0.992 to 0.999 (Table 1 and Supplementary Fig. S12).

### Expression profiles of candidate reference genes

**Figure 2:**
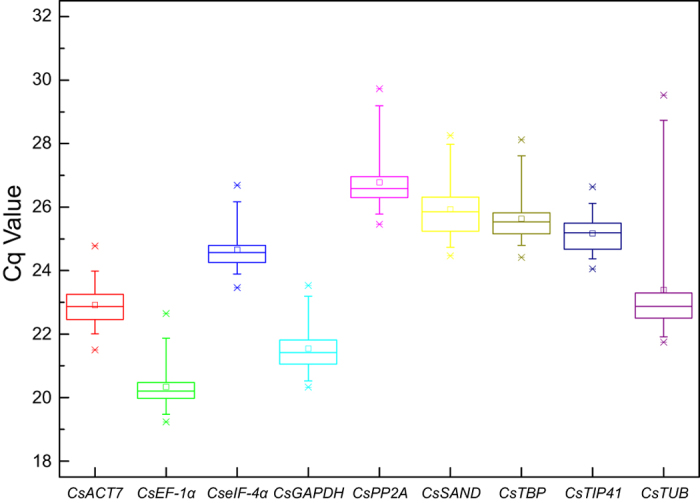
Cq values of nine candidate reference genes in all samples of *C. sinensis*. Raw Cq values of ten samples, including five developmental stages of tea plant leaves and five different hormonal stimuli, were described using a box and whiskers plot. The outer box is determined from 25th to 75th percentiles, and the inner box represents the mean value. The line across the box is the median. The whiskers represent percentiles from 5th to 95th, and outliers are depicted by asterisks.

RNA from all tea leaf samples at five developmental stages and five different hormonal stimuli were reverse transcribed into cDNA as templates for qRT-PCR detection. The expression levels of the candidate reference genes were determined by threshold cycle values (Cq) through qRT-PCR experiments (Supplementary Table S2). Cq is the amplification cycle number at which the fluorescence signal reaches above the baseline threshold. Baseline thresholds were standardized to mean 75.55. A box and whiskers plot was used to describe the raw data distribution (Fig. 2). Lower Cq values correspond to higher expression abundance, and higher Cq values correspond to lower expression abundance. At least three genes (*CsACT7*, *CsEF-1α*, and *CsGAPDH*) were highly expressed genes (19 < Cq < 25). Genes *CsPP2A*, *CsSAND*, *CsTBP*, *CsTIP41*, and *CseIF-4α* showed relatively low expression levels (23 < Cq < 30). The *CsTUB* gene (standard deviation, SD = 2.21; Cq values varied from 21.74 to 29.53) showed maximum variability, while the other eight tested genes (SD < 1.6) maintained stable expression (Supplementary Table S3).

**Figure 3:**
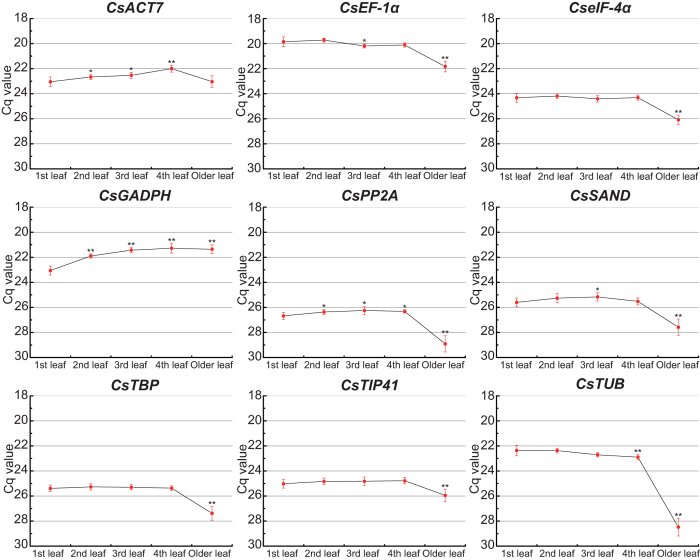
Expression profiles of candidate reference genes in various tea leaf developmental stages. Cq values in five developmental stages of tea plant leaves were counted for expression analysis. Red points represent the mean value of the Cq at a certain developmental stage. Whiskers represent the range of standard errors, and asterisks indicate significant differences in the expression levels of candidate reference genes during tea leaf development (1st leaf as the control, *P < 0.05, **P < 0.01).

The expression profiles of candidate reference genes during tea leaf developmental stages were investigated (Fig. 3). Seven genes (*CsEF-1α*, *CseIF-4α*, *CsPP2A*, *CsSAND*, *CsTBP*, *CsTIP41*, and *CsTUB*) were stably expressed in four tender leaves (1st, 2nd, 3rd, and 4th leaves) but showed significantly decreased expression levels in older leaves. The homologs of these genes in *Arabidopsis* are involved in many biological processes, such as protein translation and extension, cell signaling, and cytoskeleton formation^8^,^26^,^27^,^28^. Lower expression levels of these genes in aging tea leaves indicate that their biological functions may be partially suppressed. The general trend of expression of two other genes: *CsACT7* gene initially increased, and then decreased and restored to the original level in old leaves; *CsGAPDH* gene increasingly expressed until steadily. The *ACT7* gene in *Arabidopsis* is a basic component of the cytoskeleton and plays an essential role in germination^29^. The similar gene (*CsACT7*) of *C. sinensis* may be involved in the formation of the cytoskeleton in tender leaves; its biological function may also be partially suppressed in older leaves. The *GAPDH* gene in *Arabidopsis* is related to glycolysis, microtubule bundling, nuclear RNA transport, DNA replication, and DNA repair^30^,^31^. The *CsGAPDH* gene may be involved in a variety of biological functions during tea leaf development.

### GeNorm analysis

The expression stabilities of the nine candidate reference genes were analyzed by geNorm software^23^, which calculates gene expression stability (M) as the average pair-wise variation between all tested genes. The reference gene with the lowest M value was considered as the most stable gene. Ten experimental samples were sorted into three different subsets: “Developmental stages” (1st, 2nd, 3rd, 4th, and older leaves), “Hormonal stimuli” (ABA, GA, IAA, MeJA, and SA), and “Total” (all samples). This integration was also adopted in two other statistical algorithms (NormFinder and BestKeeper). The results of GeNorm analysis are detailed in Table 2. Nine reference genes in the three subsets showed high expression stabilities with a threshold value of below 1.5. *CseIF-4α* and *CsEF-1α* (M = 0.19) were the most stable genes, while *CsTUB* (M = 0.90) was the most variable gene in tea leaf developmental stages. Under different hormonal stimuli, *CsPP2A* and *CsTBP* (M = 0.19) were identified as the most stable genes, whereas *CsGAPDH* (M = 0.47) gene showed the worst stability. A combination of individual samples in total showed that *CseIF-4α* and *CsEF-1α* (M = 0.20) featured the most stable expression whereas the most variable genes included *CsGAPDH* (M = 0.59) and *CsTUB* (M = 0.78).

**Figure 4:**
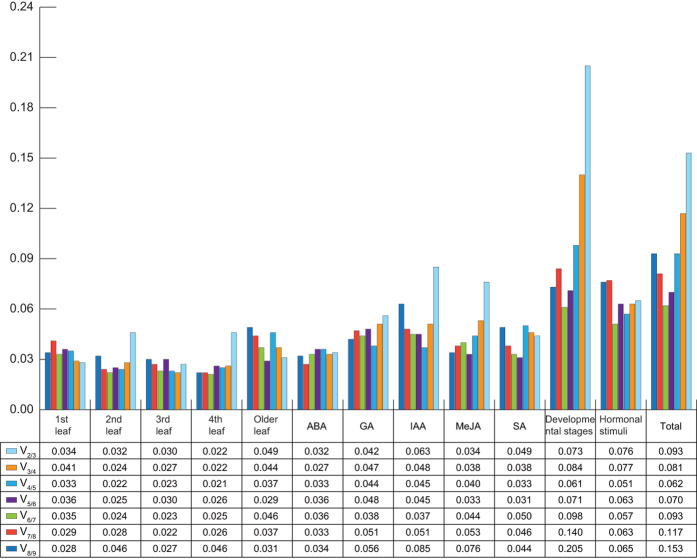
Pairwise variation (V) of candidate reference genes calculated by geNorm. Pairwise variation (V_n/n + 1_) was analyzed between the normalization factors (i.e., NF_n_ and NF_n + 1_) to determine the optimal number of reference genes. “Developmental stages” include the 1st, 2nd, 3rd, 4th, and older leaves. “Hormonal stimuli” includes ABA (abscisic acid), GA (gibberellins), IAA (indole-3-acetic acid), MeJA (methyl jasmonate), and SA (salicylic acid). “Total” represents all samples.

To determine the optimal number of reference genes for accurate normalization, pairwise variations (V_n/n + 1_, where n corresponds to the number of reference genes used to normalize gene expression) between two sequential normalization factors (NF_n_ and NF_n + 1_) were calculated by the geNorm program. A large pairwise variation (the recommended cut-off value ≥0.15) with a significant effect means that the added gene is preferably included for calculation of a reliable normalization factor; an extra reference gene is not required for normalization when the cut-off value is below 0.15. As shown in Fig. 4, the pairwise variations of all experimental samples at the V_2/3_ value were below 0.15, which indicates that two reference genes were sufficient for accurate normalization; addition of the third reference gene showed no significant effect on the results.

### NormFinder analysis

The stability value of each candidate reference gene was also calculated by NormFinder software^24^, which is based on a mathematical model of separate analysis of sample subgroups and estimation of both intra- and intergroup expression variations. Genes with more stable expression were indicated by lower average expression stability values. The results of NormFinder analysis showed that the stability ranking of the nine genes of interest was relatively consistent with the data array of geNorm (Table 2). In NormFinder analysis, the most stable reference gene for the tea leaf developmental stages was *CsTBP*; *CsEF-1α*, *CsTBP*, and *CseIF-4α*, which featured identical scores, were the most stable genes under different hormonal stimuli. Finally, the most stable reference genes in total were *CsTBP* and *CseIF-4α*, which also showed identical scores. The two worst stable genes were, *CsTUB* and *CsGAPDH*, were consistently found in the three subsets.

### BestKeeper analysis

BestKeeper software^25^ was applied as an expression standard of reference genes according to the ranking of the standard deviation (SD [±Cq]) and coefficient of variance (CV [%Cq]) of Cq values. The most stable reference genes present the lowest coefficient of variance and standard deviation (CV ± SD). SD values less than 1 were considered an acceptable range of variation^25^. The analysis results of BestKeeper are also listed in Table 2. Eight reference genes showed remarkably stable expression in all subsets, while the *CsTUB* gene with CV ± SD values of 7.94 ± 1.89 in “Developmental stages” and 4.89 ± 1.14 in “Total” was considered unacceptable for gene expression normalization. The rankings of Bestkeeper analysis revealed that the most stably expressed genes were *CsACT7* (CV ± SD = 1.84 ± 0.42) and *CsTIP41* (CV ± SD = 1.81 ± 0.45) for tea leaf developmental stages; this finding contrasts the result that the *CsACT7* gene is not a good reference gene in geNorm and NormFinder. *CseIF-4α* (CV ± SD = 1.29 ± 0.32) and *CsEF-1α* (CV ± SD = 1.69 ± 0.34) showed the most stable expression under different hormonal stimuli, and *CseIF-4α* (CV ± SD = 1.82 ± 0.45) and *CsTIP41* (CV ± SD = 1.77 ± 0.45) showed the highest expression stabilities in the total subset. Integrating the outcome of above three programs (geNorm, NormFinder, and Bestkeeper), *CsTBP* and *CseIF-4α* appear to be the most suitable reference genes in the tea developmental stages, *CsTBP* may be best reference gene under hormone treatments, and *CsTBP* and *CseIF-4α* may be used as the most suitable reference genes in total sample.

### Reference gene validation

**Figure 5:**
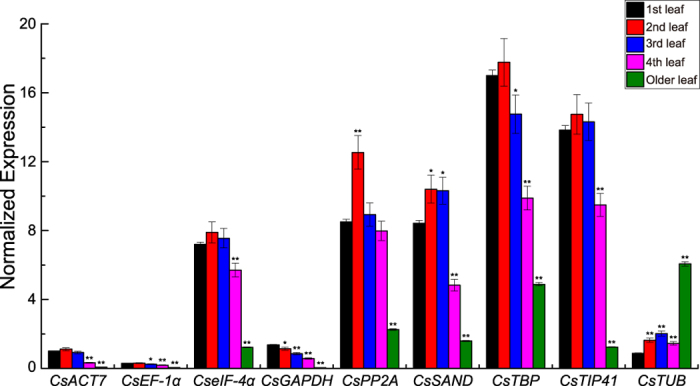
Relative quantification of *CsNAM* gene expression using candidate nine reference genes for normalization during tea leaf developmental stages in *C. sinensis*. Results were normalized against the candidate reference genes of *C. sinensis*. Asterisks indicate significant differences in the expression levels of the *CsNAM* gene during tea leaf development (1st leaf as the control, *P < 0.05, **P < 0.01).

The *NAM* gene belonging to the NAC transcription factor gene family is related to tissue development and stress signal transduction^32^,^33^. To validate the reliability of candidate reference genes used in tea leaf development, the relative expression of the tea plant *CsNAM* gene^34^ during five tea leaf development stages was normalized by using nine internal control genes (*CsACT7*, *CsEF-1α*, *CseIF-4α, CsGAPDH*, *CsPP2A*, *CsSAND*, *CsTBP*, *CsTIP41*, and *CsTUB*) (Fig. 5). If statistical insignificance is not ignored, the expression levels of the *CsNAM* gene first increased regularly in the 2nd leaf and then decreased from the 3rd to older leaves when seven internal control genes (*CsACT7*, *CsEF-1α*, *CseIF-4α, CsPP2A*, *CsSAND*, *CsTBP*, and *CsTIP41*) were used for normalization. Some discrepancies were observed in the normalization of the reference genes. The *CsNAM* gene was significantly up-regulated in the 2nd leaf when normalized by genes *CsPP2A* and *CsSAND*. The *CsNAM* gene in the 3rd leaf was significantly up-regulated and down-regulated when normalized by genes *CsSAND* and *CsTBP*, respectively. The *CsNAM* gene showed no significant difference in the 4th leaf when normalized by *CsPP2A* gene. The main discrepancies were observed during normalization of the worst reference genes, *CsGAPDH* and *CsTUB*. The *CsNAM* gene was significantly down-regulated in the 2nd leaf when normalized by the *CsGAPDH* gene. The up-regulated expression of the *CsGAPDH* gene in the 2nd leaf may lead to this outcome. When using the reference gene *CsTUB*, the *CsNAM* gene showed significant ultra-high expression in older leaf.

**Figure 6:**
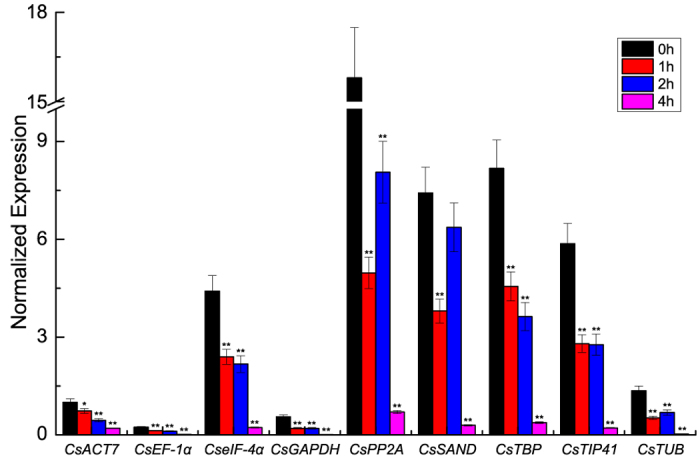
Relative quantification of *CsNAM* gene expression under GA treatment using nine candidate reference genes for normalization in *C. sinensis*. Results were normalized against the candidate reference genes of *C. sinensis*. Asterisks indicate significant differences in the expression levels of *CsNAM* gene under GA treatment (0 h treatment as the untreated control, *P < 0.05, **P < 0.01).

Normalization of the relative expression of the tea plant *CsNAM* gene under GA treatment was performed by using nine candidate reference genes to validate the reliability of candidate reference genes used for hormonal stimulation (Fig. 6). The main divergence of results was observed in the expression levels of the *CsNAM* gene between 1 h and 2 h of GA treatment. Two cases may describe this difference: (1) the expression of the *CsNAM* gene gradually decreased from 0 h to 4 h when normalized by *CsACT7*, *CsEF-1α*, *CseIF-4α*, *CsGAPDH*, *CsTBP*, and *CsTIP41* and (2) the expression of the *CsNAM* gene first decreased at 1 h and then increased at 2 h when normalized by genes *CsPP2A*, *CsSAND*, and *CsTUB*. In the first case, *CsEF-1α*, *CseIF-4α,* and *CsTBP* were stably expressed genes in NormFinder analysis. In the second case, *CsPP2A* and *CsSAND* were stably expressed genes in geNorm analysis. However, three genes *CsPP2A*, *CsSAND*, and *CsTUB* were identified as the worst reference genes in BestKeeper. Thus, the first case is more credible and the genes *CsEF-1α*, *CseIF-4α*, and *CsTBP* are the most suitable for normalization of the *CsNAM* gene under GA treatment.

## Discussion

qRT-PCR is a revolutionary technology used for accurate and sensitive detection in gene expression assay^35^,^36^. Besides analysis of animals, yeast, and bacteria, this technology has been used identify reference genes to analyze plant gene expression, including discrepant expression during tissue development and responses to biotic and abiotic stresses^8^,^37^. Using a valid internal control gene as a reference will ensure the real-time PCR data to be reliable for target genes; the use of an invalid reference gene will result in deviation. In this study, we cloned nine common internal control genes of tea plant, i.e., *CsACT7*, *CsEF-1α*, *CseIF-4α, CsGAPDH*, *CsPP2A*, *CsSAND*, *CsTBP*, *CsTIP41*, and *CsTUB*, for expression normalization in ten different samples, including five tea leaf developmental stages and five different hormonal stimuli. This study is the first to report the systematic analysis of reference genes that can be used in tea leaf development and hormonal treatment.

During qRT-PCR analysis, stable expression and suitable expression abundance for identifying valid reference genes are reliable precondition to reduce potential risks in different experimental conditions or among diverse species^38^. For instance, the *GAPDH* gene shows high stability in *Coffea arabica* but low stability in peach; *TUB* is the most stable gene in carrot leaves but the worst stable gene in berry development^6^,^39^,^40^,^41^. Moreover, as the expression level of the *UBI-1* gene in pepper is very low, this gene has been discarded as a reference gene^42^. The *18S* gene in maize grains with excessive expression abundance is not suitable for normalization of lowly expressed genes^43^. Fortunately, nine candidate reference gene of *C. sinensis* with reasonable abundance (19 < Cq < 30) were further assessed for expression normalization.

Considering that an algorithm is one-sided for evaluating the expression stability of reference genes, many statistical approaches are usually integrated to determine the best reference genes in different experimental conditions^44^,^45^. In the present study, we employed three common statistical programs, geNorm, NormFinder, and Bestkeeper, to evaluate gene expression stability among three different subsets, namely, “Developmental stages”, “Hormonal stimuli”, and “Total”. As expected, the distinct statistical algorithms generated inconsistent stability ranking among the three subsets. The geNorm algorithm determines gene expression stability by comparing the expression ratio of pair-wise gene among test samples^23^. Co-regulated genes with similar expression profiles will obtain preferential stability ranking from geNorm, leading to an erroneous choice for normalization. By contrast, the algorithms of NormFinder and Bestkeeper are less sensitive to co-regulation^24^,^25^. To investigate whether potential co-regulation in two pairs of genes (*CseIF-4α*/*CsEF-1α* and *CsPP2A*/*CsTBP*) affects the outcome of geNorm, genes among the four genes were differently removed in geNorm. Results showed that removing the top-ranked gene *CseIF-4α* or *CsEF-1α* will change the stability ranking of gene expression; removing the top-ranked gene *CsPP2A* or *CsTBP* does not markedly change in the stability ranking of gene expression only if gene *CseIF-4α* or *CsEF-1α* is lacking. This result indicates that the outputs of the best genes, namely, *CseIF-4α* and *CsEF-1α* from geNorm may be affected by their potential co-regulation. Therefore, re-integrating the outcome of programs geNorm, NormFinder, and Bestkeeper, *CsTBP* and *CsTIP41* are the most suitable reference genes in tea leaf developmental stages, *CsTBP* is the best reference gene under different hormonal stimuli, and *CsTBP* and *CsTIP41* genes can be used as the most suitable reference genes in total sample. The worst stable gene, *CsTUB* or *CsGAPDH*, was consistent in geNorm and NormFinder. *CsTUB* gene was discarded for normalization of tea leaf developmental stages since the SD of its expression exceeded the threshold value (SD > 1) in Bestkeeper.

To validate the availability of a reference gene, the expression levels of *CsNAM* gene during various tea leaf developmental stages and under GA treatment were detected using nine candidate reference genes for normalization. For tea leaf development, the normalization results of *CsNAM* gene were more consistent when genes with stable expression were used as internal controls. The two worst reference genes, *CsGAPDH* and *CsTUB*, resulted in significant differences in the normalization of the *CsNAM* gene. For GA treatment, the expression levels of the *CsNAM* gene showed similar trends upon normalization by some stable and unstable genes. This result may be explained by the fact that several candidate genes may be suitable for normalization; the expression trend of verified genes may also make up for the defects of unstable genes during normalization. Interestingly, the verification procedures described above confirmed that the more stable *CsTBP* gene is a reliable reference gene that could be available for tea leaf development and hormonal treatments.

## Methods

### Plant material and treatments

Two-year-old cutting seedlings of tea plant (*C. sinensis* cv. ‘Longjing43’) were planted in pots containing a mixture of perlite, vermiculite, and sphagnum (ratio, 1:2:3) in a climate-controlled growth chamber programmed with 70% ± 10% relative humidity, 16 h light (25 °C) with a light intensity of 300 μmol·m^−2^·s^−1^ during the daytime, and 8 h darkness (16 °C) during the nighttime (Supplementary Fig. S1). The 1st, 2nd, 3rd, 4th, and older leaves at different levels of development were collected (Fig. 1). The 3rd leaves were selected as materials for using in hormonal treatments and untreated control to unify standards. Leaves were sprayed with 1 mM gibberellins (GA treatment), 1 mM 3-indoleacetic acid (IAA treatment), 1 mM salicylic acid (SA treatment), 1 mM methyl jasmonate (MeJA treatment), or 0.1 mM abscisic acid (ABA treatment) for 2 h^46^,^47^. While GA, IAA, SA, and MeJA were dissolved in distilled water with 2% absolute ethanol, ABA was dissolved in distilled water only. Three biological experimental replicates were performed in different pots for each treatment. Tea plant leaf materials were collected, quickly immersed in liquid nitrogen and stored at –80 °C for RNA extraction.

### RNA isolation and cDNA reverse transcription

Total RNA was extracted by using the Quick RNA Isolation Kit (Huayueyang Biotech Co., Ltd., Beijing, China). RNA concentration and purity were estimated by a Nanodrop ND 1000 spectrophotometer (Nanodrop Technologies, Wilmington, DE, USA), and RNA integrity was checked by 1.2% agarose gel electrophoresis. cDNAs (20 μL) were synthesized from 1 μg of total RNA using the PrimeScript^TM^ RT reagent Kit with gDNA Eraser (TaKaRa Biotech Co., Ltd., Dalian, China).

### Selection of candidate reference genes, primer design, and gene cloning

Nine common reference genes, namely, *ACT7*, *EF-1α*, *eIF-4α*, *GAPDH*, *PP2A*, *SAND*, *TBP*, *TIP41*, and *TUB*, were selected from *Arabidopsis* genes of the TAIR database (http://www.arabidopsis.org). In the present study, potential homologues of the nine reference genes (i.e., *CsACT7*, *CsEF-1α*, *CseIF-4α*, *CsGAPDH*, *CsPP2A*, *CsSAND*, *CsTBP*, *CsTIP41*, and *CsTUB*) used for gene expression analyses were obtained by querying the *C. sinensis* transcriptome database^20^. All primers for cloning and detection of reference genes were designed using Primer Premier 5.0 software. The primer sequences used in this study are provided in Table 1 and Supplementary Table S1. The full-length sequences of nine candidate reference genes from *C. sinensis* were cloned using 2 × *Taq* Plus Master Mix (Vazyme Biotech Co., Ltd., Nanjing, China) as the polymerase. The reaction volume for PCR amplification was 20 μL, including 10 μL of 2 × *Taq* Plus Master Mix, 7 μL of ddH_2_O, 1 μL of the template cDNA, and 1 μL of each primer (10 nmol·mL^−1^). PCR was performed as follows: 5 min at 94 °C for denaturation; 35 cycles of 30 s at 94 °C (denaturation), 30 s at 52 °C (annealing), and 90 s at 72 °C (extension); and a final step of 10 min at 72 °C for extension. PCR products were gel-purified, ligated into the pMD 19-T vector, and then transformed into *Escherichia coli*. The bacterial liquids were sequenced by GenScript Corporation (Nanjing, China).

### qRT-PCR assay

qRT-PCR reactions were performed in a 96-well plate on a real-time PCR Bio-Rad iQ5 platform (Bio-Rad Laboratories, Inc., Hercules, CA, USA) using SYBR Premix *Ex Taq* (Tli RNaseH Plus) (TaKaRa Biotech Co., Ltd., Dalian, China). The reaction mixture (20 μL) contained 10 μL of SYBR Green I Mix, 2 μL of diluted cDNA (18 × dilution), 0.4 μL of each primer (10 nmol·mL^−1^), and 7.2 μL of ddH_2_O. The amplification conditions were as follows: 95 °C for 30 s, 40 cycles at 95 °C for 10 s, and 60 °C for 20 s. Melting curves were obtained to verify primer specificity through stepwise heating of the amplicon from 65 °C to 95 °C. All qRT-PCR assays included three technical and biological replicates. Standard curves were drawn to determine the amplification efficiency (E) and correlation coefficient (R^2^) of the diluted series on the basis of the diluted cDNA series (10× , 10^2^× , 10^3^× , 10^4^× , 10^5^× , and 10^6^×  dilutions). The equation (E = (10^[−1/slope]^ – 1) × 100%) was used to calculate the PCR efficiency^48^.

### Data analysis

Cq values of fluorescence curves were obtained by standardizing the baseline threshold to mean 75.55. The raw Cq data are listed in the Supplementary Table S2. Three common software (i.e., geNorm^23^, NormFinder^24^, and BestKeeper^25^) were used to calculate the expression stability of candidate reference genes. Cq values were converted to relative quantities according to the formula: 2^−ΔCt^ (ΔCt = the corresponding Cq value – minimum Cq)^5^. geNorm and NormFinder calculations are based on these converted quantities; raw Cq values were directly analyzed by BestKeeper. Pairwise variation values were calculated by geNorm software with a recommended cut-off value ≥0.15. In addition, the *CsNAM* gene of *C. sinensis*^34^ was analyzed to validate the reliability of the candidate reference genes. The statistical tests of gene expression data used in Figs 3, 5, and  6 were calculated by one-way ANOVA. Statistical significance is considered at *P < 0.05 and **P < 0.01. Variance analyses were performed based on the EXCEL program.

## Additional Information

**How to cite this article**: Wu, Z.-J. *et al.* Selection of suitable reference genes for qRT-PCR normalization during leaf development and hormonal stimuli in tea plant (*Camellia sinensis*). *Sci. Rep.* **6**, 19748; doi: 10.1038/srep19748 (2016).

## Supplementary Material

Supporting Information

## Figures and Tables

**Table 1:** Candidate reference genes, primer sequences, and amplicon characteristics of *C. sinensis*.

Gene symbol	Gene name	*Arabidopsis* homolog gene	Primer sequence (5′–3′) forward/reverse	Amplicon length (bp)	E (%)	Tm °C
*CsACT7*	Actin7 gene	AT5G09810	TTGGATTCTGGGGATGGTGTTAGC	195	102.6	84.5
			AGCAAGTTTCTCTTTCACATCACGG			
*CsEF-1α*	Elongation factor-1α gene	AT5G60390	TTGACAAGCGTGTGATTGAGAGGT	177	107.0	84
			GGGCATCAATGACAGTGCAGTAGTA			
*CseIF-4α*	Eukaryotic translation initiation factor 4α-1 gene	AT3G13920	TGAGTTACTTGTGGCTGATGGAGAA	145	107.5	82
			CCTTTGCTGAATTGCAGACGGCTT			
*CsGAPDH*	Glyceraldehyde-3-phosphate dehydrogenase gene	AT1G42970	CCCTCTAAGTCCTCTCACTCCTCCT	158	95.5	84
			GTTGCTTGGGCAGCCACTACAT			
*CsPP2A*	Protein phosphatase 2A gene	AT3G21650	AAGAAGAGGAACTGGCGACGGAAC	153	102.9	83.5
			CAAACAGGTCCAGCAAACGCAAC			
*CsSAND*	SAND family protein gene	AT2G28390	GCCTGAACCGTCTTCTGTGGAGT	184	101.9	87
			CTCAATCTCAGACACACTGGTGCTA			
*CsTBP*	TATA-box binding protein gene	AT1G55520	GGCGGATCAAGTGTTGGAAGGGAG	166	107.0	85
			ACGCTTGGGATTGTATTCGGCATTA			
*CsTIP41*	Tap42-interacting protein of 41 kDa gene	AT4G34270	TGGAGTTGGAAGTGGACGAGACCGA	176	103.6	87
			CTCTGGAAAGTGGGATGTTTGAAGC			
*CsTUB*	Tubulin beta-6 gene	AT5G12250	AATGAGGCTTCTTGTGGGAGGTTTG	147	107.0	86.5
			GTTATTTCCAGCACCAGACTGACCG			

**Table 2:** Gene expression stability ranked by geNorm, NormFinder, and BestKeeper.

Group	Rank	geNorm		NormFinder		BestKeeper		
		*Gene*	Stability	*Gene*	Stability	*Gene*	SD [±Cq]	CV [%Cq]
Developmental stages	1	*CseIF-4α*	0.19	*CsTBP*	0.01	*CsACT7*	0.42	1.84
	2	*CsEF-1α*	0.19	*CsTIP41*	0.04	*CsTIP41*	0.45	1.81
	3	*CsTBP*	0.22	*CseIF-4α*	0.05	*CsGAPDH*	0.57	2.63
	4	*CsSAND*	0.28	*CsPP2A*	0.06	*CseIF-4α*	0.58	2.36
	5	*CsPP2A*	0.31	*CsSAND*	0.09	*CsEF-1α*	0.61	3.00
	6	*CsTIP41*	0.36	*CsEF-1α*	0.12	*CsTBP*	0.66	2.58
	7	*CsACT7*	0.46	*CsACT7*	0.16	*CsSAND*	0.75	2.91
	8	*CsGAPDH*	0.63	*CsTUB*	0.17	*CsPP2A*	0.82	3.06
	9	*CsTUB*	0.90	*CsGAPDH*	0.27	*CsTUB*	1.89	7.94
Hormone stimuli	1	*CsPP2A*	0.19	*CsEF-1α*	0.07	*CseIF-4α*	0.32	1.29
	2	*CsTBP*	0.19	*CsTBP*	0.07	*CsEF-1α*	0.34	1.69
	3	*CsSAND*	0.23	*CseIF-4α*	0.07	*CsGAPDH*	0.39	1.84
	4	*CseIF-4α*	0.28	*CsPP2A*	0.08	*CsTIP41*	0.39	1.54
	5	*CsEF-1α*	0.29	*CsSAND*	0.09	*CsTBP*	0.41	1.62
	6	*CsTUB*	0.33	*CsTIP41*	0.09	*CsACT7*	0.45	1.94
	7	*CsTIP41*	0.37	*CsACT7*	0.09	*CsPP2A*	0.47	1.77
	8	*CsACT7*	0.41	*CsTUB*	0.13	*CsTUB*	0.51	2.22
	9	*CsGAPDH*	0.47	*CsGAPDH*	0.23	*CsSAND*	0.52	1.99
Total	1	*CsEF-1α*	0.20	*CsTBP*	0.06	*CseIF-4α*	0.45	1.82
	2	*CseIF-4α*	0.20	*CseIF-4α*	0.06	*CsTIP41*	0.45	1.77
	3	*CsTBP*	0.26	*CsTIP41*	0.07	*CsEF-1α*	0.48	2.34
	4	*CsPP2A*	0.31	*CsPP2A*	0.08	*CsACT7*	0.50	2.16
	5	*CsSAND*	0.33	*CsSAND*	0.10	*CsTBP*	0.51	2.01
	6	*CsTIP41*	0.37	*CsEF-1α*	0.10	*CsGAPDH*	0.52	2.41
	7	*CsACT7*	0.46	*CsACT7*	0.14	*CsPP2A*	0.63	2.34
	8	*CsGAPDH*	0.59	*CsTUB*	0.15	*CsSAND*	0.67	2.57
	9	*CsTUB*	0.78	*CsGAPDH*	0.26	*CsTUB*	1.14	4.89

SD [±Cq]: standard deviation of the Cq; CV [%Cq]: coefficient of variance expressed as a percentage of the Cq level.
