# Supplementary material for: Selection of suitable reference genes for qRT-PCR normalization during leaf development and hormonal stimuli in tea plant (Camellia sinensis)
Source: Sci Rep. 2016 Jan 27;6:19748. doi: 10.1038/srep19748 (PMC4728435; doi:10.1038/srep19748)
Supplement: Supporting Information [file srep19748-s1.pdf]

Selection of suitable reference genes for qRT-PCR normalization during leaf development and hormonal stimuli in tea plant (*Camellia sinensis*)

Zhi-Jun Wu[1], Chang Tian[2], Qian Jiang[2], Xing-Hui Li[1], Jing Zhuang[1]*

[1]*Tea Science Research Institute, College of Horticulture, Nanjing Agricultural University, Nanjing, 210095, China*

[2]*State Key Laboratory of Crop Genetics and Genetics and Germplasm Enhancement, College of Horticulture. Nanjing Agricultural University, Nanjing, 210095, China*

*Please address all correspondence to: J. Zhuang (zhuangjing@njau.edu.cn)

Dr. Jing Zhuang

Professor

Tea Science Research Institute,

College of Horticulture,

Nanjing Agricultural University,

Nanjing, 210095, China

Fax: 86 25 84395182

Email: zhuangjing@njau.edu.cn

Running title: *Suitable reference genes in tea plant*

Supporting Information

Figure S1. Photograph of *C. sinensis* cv. ‘Longjing43’.

The tea plants of ‘Longjing43’ are two-year-old cutting seedlings.

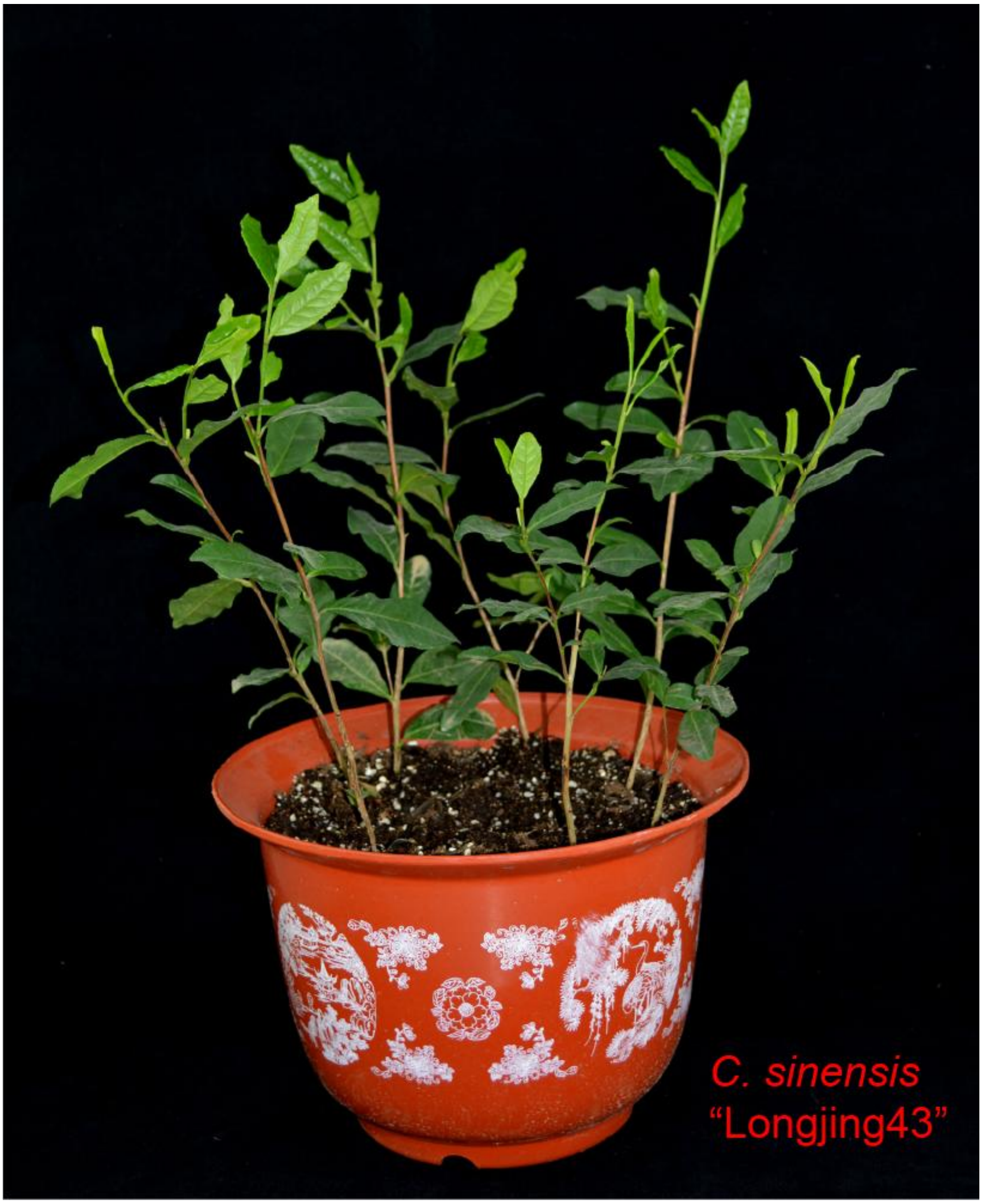

Figure S2. Nucleotide and deduced amino acid sequences of *CsACT7* from *C. sinensis*.

```
1       ATGGCTGATGCTGAGGATATTCAGCCCCTTGTCTGTGACAATGGAACTGGAATGGTGAAGGCTGGGTTTGCT
         M  A  D  A  E  D  I  Q  P  L  V  C  D  N  G  T  G  M  V  K  A  G  F  A
73      GGTGATGATGCTCCCAGGGCAGTTTTCCCCAGTATTGTAGGTCGACCCAGGCACACTGGTGTCATGGTTGGG
         G  D  D  A  P  R  A  V  F  P  S  I  V  G  R  P  R  H  T  G  V  M  V  G
145     ATGGGCCAGAAAGATGCTTATGTAGGTGATGAAGCCCAATCCAAAAGAGGTATTCTTACCTTGAAATACCCA
         M  G  Q  K  D  A  Y  V  G  D  E  A  Q  S  K  R  G  I  L  T  L  K  Y  P
217     ATTGAACATGGCATTGTCAGCAATTGGGATGACATGGAGAAGATCTGGCATCATACTTTCTATAACGAGCTC
         I  E  H  G  I  V  S  N  W  D  D  M  E  K  I  W  H  H  T  F  Y  N  E  L
289     CGTGTTGCCCCTGAAGAGCACCCAGTACTTCTTACAGAAGCACCACTGAACCCGAAGGCAAATAGGGAGAAG
         R  V  A  P  E  E  H  P  V  L  L  T  E  A  P  L  N  P  K  A  N  R  E  K
361     ATGACTCAAATTATGTTTGAGACCTTCAATGTGCCCGCCATGTATGTTTCTATCCAGGCAGTTCTGTCTCTT
         M  T  Q  I  M  F  E  T  F  N  V  P  A  M  Y  V  S  I  Q  A  V  L  S  L
433     TATGCCAGTGGTCGTACAACAGGTATTGTCTTGGATTCTGGGGATGGTGTTAGCCACACAGTGCCAATTTAC
         Y  A  S  G  R  T  T  G  I  V  L  D  S  G  D  G  V  S  H  T  V  P  I  Y
505     GAAGGTTATACCCTCCCACATGCAATCCTTCGGTTGGACCTTGCTGGTCGTGATCTAACAGATTCATTGATG
          E  G  Y  T  L  P  H  A  I  L  R  L  D  L  A  G  R  D  L  T  D  S  L  M
577     AAGATCCTCACTGAGCGAGGGTACTCCTTCACCACAACAGCTGAACGGGAAATTGTCCGTGATGTGAAAGAG
         K  I  L  T  E  R  G  Y  S  F  T  T  T  A  E  R  E  I  V  R  D  V  K  E
649     AAACTTGCTTATGTTGCCCTTGATTACGAGCAGGAGTTGGAAACTGCAAAGAGCAGCTCTGCAGTGGAGAAA
         K  L  A  Y  V  A  L  D  Y  E  Q  E  L  E  T  A  K  S  S  S  A  V  E  K
721     AGCTATGAACTTCCTGATGGGCAAGTGATCACTATCGGAGCAGAGAGATTCCGCTGCCCTGAAGTTCTATTC
         S  Y  E  L  P  D  G  Q  V  I  T  I  G  A  E  R  F  R  C  P  E  V  L  F
793     CAGCCATCGCTCATTGGAATGGAAGTTGCTGGAATCCACGAATCCACCTACAACTCCATCATGAAATGTGAT
         Q  P  S  L  I  G  M  E  V  A  G  I  H  E  S  T  Y  N  S  I  M  K  C  D
865     GTTGATATCAGGAAGGATCTCTATGGAAACATTGTCCTTAGTGGTGGATCAACCATGTTCCCAGGGATTGCT
         V  D  I  R  K  D  L  Y  G  N  I  V  L  S  G  G  S  T  M  F  P  G  I  A
937     GACCGGATGAGCAAGGAAATCACTGCCCTTGCTCCTAGTAGCATGAAGATCAAAGTGGTGGCACCTCCTGAG
         D  R  M  S  K  E  I  T  A  L  A  P  S  S  M  K  I  K  V  V  A  P  P  E
1009    AGAAAGTACAGTGTCTGGATTGGAGGCTCAATCTTGGCATCTCTCAGCACATTCCAGCAGATGTGGATATCC
         R  K  Y  S  V  W  I  G  G  S  I  L  A  S  L  S  T  F  Q  Q  M  W  I  S
1081    AAGGATGAATATGATGAATCTGGTCCAGCCATTGTCCACAGGAAGTGCTTTTGA
         K  D  E  Y  D  E  S  G  P  A  I  V  H  R  K  C  F  *
```

Figure S3. Nucleotide and deduced amino acid sequences of *CsEF-1α* from *C. sinensis*.

```
1       ATGGGTAAGGAAAAAGTTCACATTAACATTGTGGTCATTGGCCATGTCGACTCTGGGAAGTCAACCACCACT
         M  G  K  E  K  V  H  I  N  I  V  V  I  G  H  V  D  S  G  K  S  T  T  T
73      GGTCATCTCATCTACAAGCTTGGAGGTATTGACAAGCGTGTGATTGAGAGGTTTGAGAAGGAGGCTGCTGAG
         G  H  L  I  Y  K  L  G  G  I  D  K  R  V  I  E  R  F  E  K  E  A  A  E
145     ATGAACAAACGTTCATTCAAGTATGCCTGGGTCTTGGACAAGCTCAAGGCTGAACGTGAACGTGGTATTACC
         M  N  K  R  S  F  K  Y  A  W  V  L  D  K  L  K  A  E  R  E  R  G  I  T
217     ATTGATATTGCTTTGTGGAAGTTTGAGACCACCAAGTACTACTGCACTGTCATTGATGCCCCTGGCCATCGT
         I  D  I  A  L  W  K  F  E  T  T  K  Y  Y  C  T  V  I  D  A  P  G  H  R
289     GACTTTATCAAGAACATGATCACTGGTACCTCACAGGCTGACTGTGCAGTCCTTATCATTGACTCAACAACT
         D  F  I  K  N  M  I  T  G  T  S  Q  A  D  C  A  V  L  I  I  D  S  T  T
361     GGTGGGTTTGAAGCTGGTATTTCCAAGGATGGTCAGACACGTGAGCATGCTTTGCTTGCCTTTACCCTTGGT
         G  G  F  E  A  G  I  S  K  D  G  Q  T  R  E  H  A  L  L  A  F  T  L  G
433     GTCAAGCAAATGATCTGCTGTTGCAACAAGATGGATGCAACAACCCCAAAGTATTCAAAGGCAAGGTATGAT
         V  K  Q  M  I  C  C  C  N  K  M  D  A  T  T  P  K  Y  S  K  A  R  Y  D
505     GAAATTGTTAAGGAAGTGTCTTCCTATCTGAAGAAGGTGGGTTATAACCCTGACAAGATCCCCTTTGTCCCA
         E  I  V  K  E  V  S  S  Y  L  K  K  V  G  Y  N  P  D  K  I  P  F  V  P
577     ATCTCTGGATTTGAGGGTGACAACATGATTGAAAGGTCAACCAACCTTGACTGGTACAAGGGCCCAACCCTG
         I  S  G  F  E  G  D  N  M  I  E  R  S  T  N  L  D  W  Y  K  G  P  T  L
649     CTCGATGCCCTTGACATGATTTCGGAACCCAAGAGGCCCTCGGACAAGCCTCTCCGTCTCCCACTTCAGGAC
         L  D  A  L  D  M  I  S  E  P  K  R  P  S  D  K  P  L  R  L  P  L  Q  D
721     GTCTACAAGATTGGTGGCATTGGCACTGTCCCTGTTGGCCGTGTTGAGACTGGTTTAATCAAACCCGGCATG
         V  Y  K  I  G  G  I  G  T  V  P  V  G  R  V  E  T  G  L  I  K  P  G  M
793     GTTGTCACTTTTGGCCCAACTGGTCTCACCACTGAAGTTAAGTCAGTAGAAATGCACCACGAGGCTCTCCTG
         V  V  T  F  G  P  T  G  L  T  T  E  V  K  S  V  E  M  H  H  E  A  L  L
865     GAGGCCCTACCAGGCGACAATGTTGGTTTCAATGTAAAAAATGTTGCTGTCAAGGATCTCAAACGGGGGTTT
         E  A  L  P  G  D  N  V  G  F  N  V  K  N  V  A  V  K  D  L  K  R  G  F
937     GTTGCCTCAAACTCCAAGGACGATCCTGCAAAGGAAGCTGCCAACTTTACTTCCCAGGTCATTATCATGAAC
         V  A  S  N  S  K  D  D  P  A  K  E  A  A  N  F  T  S  Q  V  I  I  M  N
1009    CATCCTGGCCAAATTGGAAATGGTTATGCCCCAGTTCTCGATTGCCACACTTCCCACATTGCAGTCAAATTT
         H  P  G  Q  I  G  N  G  Y  A  P  V  L  D  C  H  T  S  H  I  A  V  K  F
1081    GCTGAGATTCTGACCAAGATTGACAGGAGGTCTGGGAAGGAGCTCGAGAAGGAGCCCAAATTCTTGAAGAAC
         A  E  I  L  T  K  I  D  R  R  S  G  K  E  L  E  K  E  P  K  F  L  K  N
1153    GGTGATGCTGGGATGGTGAAGATGATTCCAACAAAGCCCATGGTGGTGGAGACTTTTTCCGAGTACCCACCA
         G  D  A  G  M  V  K  M  I  P  T  K  P  M  V  V  E  T  F  S  E  Y  P  P
1225    CTTGGTCGTTTTGCTGTGAGAGACATGCGTCAAACTGTTGCTGTTGGTGTCATCAAGAGTGTTGAGAAGAAG
         L  G  R  F  A  V  R  D  M  R  Q  T  V  A  V  G  V  I  K  S  V  E  K  K
1297    GATCCATCTGGTGCCAAGGTCACCAAGGCTGCTCTGAAGAAGAAATGA
         D  P  S  G  A  K  V  T  K  A  A  L  K  K  K  *
```

Figure S4. Nucleotide and deduced amino acid sequences of *CseIF-4α* from *C. sinensis*.

```
1       ATGGCTGGCTTGGCACCTGAGGGTTCTCAATTTGATGCTCGTCAATTTGATGCCAAAATGAATGAGTTACTT
         M  A  G  L  A  P  E  G  S  Q  F  D  A  R  Q  F  D  A  K  M  N  E  L  L
73      GTGGCTGATGGAGAAGAATTCTTCACCTCATATGACGAGGTTTATGAAAGCTTTGATGCAATGGGCTTACAA
         V  A  D  G  E  E  F  F  T  S  Y  D  E  V  Y  E  S  F  D  A  M  G  L  Q
145     GAAAATCTCCTGAGGGGCATCTATGCTTATGGTTTTGAGAAGCCGTCTGCAATTCAGCAAAGGGGGATAGTT
         E  N  L  L  R  G  I  Y  A  Y  G  F  E  K  P  S  A  I  Q  Q  R  G  I  V
217     CCTTTCTGCAAAGGACTTGATGTAATTCAACAAGCACAGTCTGGAACTGGAAAAACTGCAACATTCTGCTCT
         P  F  C  K  G  L  D  V  I  Q  Q  A  Q  S  G  T  G  K  T  A  T  F  C  S
289     GGAATTCTGCAGCAGCTTGATTATAGCTTGGTTGAGTGCCAAGCTTTGGTTCTTGCACCCACTCGTGAACTT
         G  I  L  Q  Q  L  D  Y  S  L  V  E  C  Q  A  L  V  L  A  P  T  R  E  L
361     GCCCAGCAAATTGAGAAGGTTATGCGAGCACTAGGTGTCTATCTTGGTGTGAAGGTTCATGCTTGTGTTGGA
         A  Q  Q  I  E  K  V  M  R  A  L  G  V  Y  L  G  V  K  V  H  A  C  V  G
433     GGGACTAGTGTGCGCGAAGATCAGCGCATTCTCTCAAGTGGGGTTCATGTTGTTGTTGGTACTCCTGGTCGT
         G  T  S  V  R  E  D  Q  R  I  L  S  S  G  V  H  V  V  V  G  T  P  G  R
505     GTGTTTGACATGTTGCGGAGACAATCACTTCGCCCTGATTGCATCAAAATGTTTGTCTTGGATGAAGCTGAT
         V  F  D  M  L  R  R  Q  S  L  R  P  D  C  I  K  M  F  V  L  D  E  A  D
577     GAAATGCTGTCACGAGGTTTTAAAGATCAGATCTATGATATTTTCCAGTTGCTGCCACCCAAAATCCAGGTT
         E  M  L  S  R  G  F  K  D  Q  I  Y  D  I  F  Q  L  L  P  P  K  I  Q  V
649     GGGGTGTTCTCTGCCACAATGCCACCAGAGGCTCTTGAAATCACCAGGAAATTCATGAATAAGCCTGTGAGG
         G  V  F  S  A  T  M  P  P  E  A  L  E  I  T  R  K  F  M  N  K  P  V  R
721     ATTCTTGTGAAACGTGACGAACTTACTCT*GAGGGTATCAAGCAATTTTATGTGAATGTTGACAAGGAGGAA
         I  L  V  K  R  D  E  L  T  *  E  G  I  K  Q  F  Y  V  N  V  D  K  E  E
793     TGGAAGCTTGAGACACTTTGTGATCTCTATGAGACCTTGGCCATAACCCAAAGCGTCATCTTTGTTAACACC
         W  K  L  E  T  L  C  D  L  Y  E  T  L  A  I  T  Q  S  V  I  F  V  N  T
865     CGACGCAAGGTTGACTGGCTCACTGACAAAATGCGCAGCCGTGATCACACGGTATCTGCTACCCATGGAGAC
         R  R  K  V  D  W  L  T  D  K  M  R  S  R  D  H  T  V  S  A  T  H  G  D
937     ATGGATCAGAACACTAGAGATATCATTATGCGGGAATTCCGGTCTGGTTCCTCTCGTGTGCTTATCACAACT
         M  D  Q  N  T  R  D  I  I  M  R  E  F  R  S  G  S  S  R  V  L  I  T  T
1009    GATCTTTTGGCTCGTGGTATTGATGTCCAGCAAGTCTCTCTTGTGATAAATTATGATCTGCCAACCCAACCT
         D  L  L  A  R  G  I  D  V  Q  Q  V  S  L  V  I  N  Y  D  L  P  T  Q  P
1081    GAGAACTACCTTCATCGAATTGGGCGTAGTGGAAGGTTTGGGAGGAAGGGTGTTGCCATCAACTTTGTGACC
         E  N  Y  L  H  R  I  G  R  S  G  R  F  G  R  K  G  V  A  I  N  F  V  T
1153    AAAGATGATGAAAGGATGCTGGCTGACATCCAGAGGTTCTATAATGTGGTAGTTGAGGAGCTCCCAGCAAAT
         K  D  D  E  R  M  L  A  D  I  Q  R  F  Y  N  V  V  V  E  E  L  P  A  N
1225    GTTGCTGATCTCCTTTGA
         V  A  D  L  L  *
```

Figure S5. Nucleotide and deduced amino acid sequences of *CsGAPDH* from *C. sinensis*.

```
1     ATGGCCACCCATGCTGCTCTGGCTTCTTCAAGAATCCCATCTCACACCTCGCTACTTCCCTCTAAGTCCTCT
       M  A  T  H  A  A  L  A  S  S  R  I  P  S  H  T  S  L  L  P  S  K  S  S
73    CACTCCTCCTGCTTCCCCACTCAATGCCTTTCCAAGAGACTGGAAGTAGCAGAGTTTTCTGGTCTTCGATCC
       H  S  S  C  F  P  T  Q  C  L  S  K  R  L  E  V  A  E  F  S  G  L  R  S
145   AGTGGACGTGTGACATACGCCAAGAATGCTAGGGAAGCATCCTTGTTTGATGTAGTGGCTGCCCAAGCAACT
       S  G  R  V  T  Y  A  K  N  A  R  E  A  S  L  F  D  V  V  A  A  Q  A  T
217   CCCATGACTGCAGGTTCAACCCCTGTCAAGGGACAAACAGTGGCCAAATTAAAGGTAGCAATTAATGGTTTT
       P  M  T  A  G  S  T  P  V  K  G  Q  T  V  A  K  L  K  V  A  I  N  G  F
289   GGACGCATTGGCCGCAACTTTCTCCGGTGCTGGCATGGCCGGAAGAACTCCCCCGTTGATGTTATTGTGGTT
       G  R  I  G  R  N  F  L  R  C  W  H  G  R  K  N  S  P  V  D  V  I  V  V
361   AACGACAGTGGTGGTGTCAAGAATGCATCTCACTTGCTGAAGTATGATTCATTGTTGGGCACTTTCAAAGCA
       N  D  S  G  G  V  K  N  A  S  H  L  L  K  Y  D  S  L  L  G  T  F  K  A
433   GAAGTGAAAATAGTGGACAATGAGACCATCAGTGTTGATGGTAAGCCCATCAAAGTTGTCTCTAGTAGGGAC
       E  V  K  I  V  D  N  E  T  I  S  V  D  G  K  P  I  K  V  V  S  S  R  D
505   CCTTTGAAGCTCCCTTGGGCTGAACTAGGCATTGACATTGTTATTGAGGGGACAGGGGTGTTTGTGGATGGA
       P  L  K  L  P  W  A  E  L  G  I  D  I  V  I  E  G  T  G  V  F  V  D  G
577   CCTGGGGCTGGGAAGCACATCCAAGCTGGTGCCAAGAAAGTTATTATCACTGCTCCAGCAAAAGGTGCCGAC
       P  G  A  G  K  H  I  Q  A  G  A  K  K  V  I  I  T  A  P  A  K  G  A  D
649   ATTCCCACCTATGTTGTTGGAGTCAATGAAGGAGACTACGCCCATGATGTTTCTAACATTGTAAGCAATGCT
       I  P  T  Y  V  V  G  V  N  E  G  D  Y  A  H  D  V  S  N  I  V  S  N  A
721   TCTTGCACCACAAACTGTTTAGCTCCTTTCGTGAAAGTCTTGGATGAAGAACTCGGTATTGTCAAGGGGACC
       S  C  T  T  N  C  L  A  P  F  V  K  V  L  D  E  E  L  G  I  V  K  G  T
793   ATGACCACCACTCATTCCTACACTGGAGACCAGAGACTCTTAGATGCTTCACACCGGGACTTGAGGCGAGCC
       M  T  T  T  H  S  Y  T  G  D  Q  R  L  L  D  A  S  H  R  D  L  R  R  A
865   AGAGCTGCAGCACTTAACATAGTCCCAACAAGCACCGGTGCAGCCAAGGCCGTGTCTCTAGTGCTTCCACAG
       R  A  A  A  L  N  I  V  P  T  S  T  G  A  A  K  A  V  S  L  V  L  P  Q
937   CTCAAGGGCAAGCTCAACGGCATTGCTCTCCGTGTTCCCACACCAAATGTATCTGTCGTTGATCTTGTTGTG
       L  K  G  K  L  N  G  I  A  L  R  V  P  T  P  N  V  S  V  V  D  L  V  V
1009  AACGTCGCGAAAAAAGGAATATCTGCTGAAGACGTTAATGCTGCCTTTAGAAAAGCAGCCGATGGACCATTG
       N  V  A  K  K  G  I  S  A  E  D  V  N  A  A  F  R  K  A  A  D  G  P  L
1081  AAGGGTATATTAGCCGTGTGTGATGTCCCTCTTGTGTCAGTCGATTTCAGGTGCTCTGATGTTTCTTCCACC
       K  G  I  L  A  V  C  D  V  P  L  V  S  V  D  F  R  C  S  D  V  S  S  T
1153  ATCGATTCATCTTTGACAATGGTCATGGGAGACGACATGGTCAAGGTAGTGGCATGGTACGACAATGAATGG
       I  D  S  S  L  T  M  V  M  G  D  D  M  V  K  V  V  A  W  Y  D  N  E  W
1225  GGATACAGCCAACGTGTTGTCGATTTGGCACATTTGGTAGCAAGCAAGTGGCCAGGCATGCCTGCACAAGGA
       G  Y  S  Q  R  V  V  D  L  A  H  L  V  A  S  K  W  P  G  M  P  A  Q  G
1297  AGTGGAGATCCATTGGAGGATTTTTGCGAGACAAGCCCTGCTGAGAAGGAGTGCAAGGTTTATGAAGCTTAA
       S  G  D  P  L  E  D  F  C  E  T  S  P  A  E  K  E  C  K  V  Y  E  A  *
```

Figure S6. Nucleotide and deduced amino acid sequences of *CsPP2A* from *C. sinensis*.

```
1     ATGATTAGGCAGATTCTGGGTAAGCTTCCTCGGAAGCCATCGTCGAAATCATCACACAACGATTCGAACAAT
       M  I  R  Q  I  L  G  K  L  P  R  K  P  S  S  K  S  S  H  N  D  S  N  N
73    GATGGGGCGGTTTCAACTATGAATTCCTCGACTTGGAATTCCATGAATAGCAGCTCGAAAAGCACTACGGTT
       D  G  A  V  S  T  M  N  S  S  T  W  N  S  M  N  S  S  S  K  S  T  T  V
145   TCTGGGAAAGTTTCGAATTTGGGTTCTGGTGCAGGTCGCTCTATCAATGGAACATATGCCCCAAACTCAATG
       S  G  K  V  S  N  L  G  S  G  A  G  R  S  I  N  G  T  Y  A  P  N  S  M
217   AGTAAATCAAATCAAGGGAAGAAATCGGGTCCCTTGGCTGCTCAAGGTGGTGGACCGGTGTCGAATATTGCA
       S  K  S  N  Q  G  K  K  S  G  P  L  A  A  Q  G  G  G  P  V  S  N  I  A
289   ACATATGAAGCTTTGCCAAGTTTTCGTGATGTTCCAAACCCAGAAAAGCAGAATCTTTTCATTAGGAAGTTG
       T  Y  E  A  L  P  S  F  R  D  V  P  N  P  E  K  Q  N  L  F  I  R  K  L
361   AAAATGTGTTGTGTTGTCTTTGATTTTAGTGACCCCTCTAAGAATATCAAAGAGAAGGATATAAAGCGGCAG
       K  M  C  C  V  V  F  D  F  S  D  P  S  K  N  I  K  E  K  D  I  K  R  Q
433   ACATTGCTTGAGCTTGTTGATTATATCTCCACTGTAAATTCAAAGTTCAATGAGGTCACAATGCAGGAAATT
       T  L  L  E  L  V  D  Y  I  S  T  V  N  S  K  F  N  E  V  T  M  Q  E  I
505   ACAAAAATGGTAGCTACCAATTTATTCAGAACTTTTCTATCCACTAATCATGAAAGCAAGTCACCTGATATG
       T  K  M  V  A  T  N  L  F  R  T  F  L  S  T  N  H  E  S  K  S  P  D  M
577   TATGATGCAGAAGAAGAGGAACTGGCGACGGAACCATCGTGGCCTCATCTTCAGATTGTGTATGAATTTCTA
       Y  D  A  E  E  E  E  L  A  T  E  P  S  W  P  H  L  Q  I  V  Y  E  F  L
649   CTTAGATTTGTGGCTTCGTCAGAGACAGATGCCAAGCTTGCTAAAAGATATGTAGACCATTCGTTTGTGTTG
       L  R  F  V  A  S  S  E  T  D  A  K  L  A  K  R  Y  V  D  H  S  F  V  L
721   CGTTTGCTGGACCTGTTTGACTCTGAGGATCAAAGAGAGAGGGAGTACTTGAAGACGATTCTCCACCGCATT
       R  L  L  D  L  F  D  S  E  D  Q  R  E  R  E  Y  L  K  T  I  L  H  R  I
793   TATGGGAAGTTTATGGTGCATCGGCCATACATTAGGAAAGCCATCAACAATATCTTCTACCGTTTTATCTTT
       Y  G  K  F  M  V  H  R  P  Y  I  R  K  A  I  N  N  I  F  Y  R  F  I  F
865   GAGACAGAGAAGCACAATGGGATAGCAGAATTGCTTGAGATCTTGGGCAGTATTATTAATGGTTTTGCTTTG
       E  T  E  K  H  N  G  I  A  E  L  L  E  I  L  G  S  I  I  N  G  F  A  L
937   CCTTTAAAGGAAGAGCACAAGCTTTTCCTTGTCCGTGCCTTGATTCCACTTCACAAGCCCAAGTGTGTAGCC
       P  L  K  E  E  H  K  L  F  L  V  R  A  L  I  P  L  H  K  P  K  C  V  A
1009  ATGTATCACCAACAACTTTCATATTGCATCACTCAGTTTGTGGAGAAGGATATAAAGCTAGCTGACACGGTA
       M  Y  H  Q  Q  L  S  Y  C  I  T  Q  F  V  E  K  D  I  K  L  A  D  T  V
1081  ATTCGTGGTCTTTTAAAGTATTGGCCATTAACTAACAGTTCAAAGGAGGTGATGTTCCTCGGTGAGTTGGAA
       I  R  G  L  L  K  Y  W  P  L  T  N  S  S  K  E  V  M  F  L  G  E  L  E
1153  GAAGTTCTAGAAGCCACACAGGCTGCAGAATTTCAGCGCTGCATGGTCCCCCTTTTCCGTCAGATTGGACGC
       E  V  L  E  A  T  Q  A  A  E  F  Q  R  C  M  V  P  L  F  R  Q  I  G  R
1225  TGCCTCAATAGCTTACATTTTCAGGTGGCTGAGCGTGCCTTGTTTCTCTGGAACAACGATCACATTGGGAAT
       C  L  N  S  L  H  F  Q  V  A  E  R  A  L  F  L  W  N  N  D  H  I  G  N
1297  TTGATCACGCAGAACCGTAAAGTAATACTGCCTATAATTTTCCCAGCCTTGGAGAGAAACACACGAAGTCAC
       L  I  T  Q  N  R  K  V  I  L  P  I  I  F  P  A  L  E  R  N  T  R  S  H
1369  TGGAACCAAGCTGTTCAGAGCCTGACACTTAATGTGAGGAAGATATTTTCAGATGCTGACCAAGCACTGTTT
       W  N  Q  A  V  Q  S  L  T  L  N  V  R  K  I  F  S  D  A  D  Q  A  L  F
1441  GATGAGTGCCTAGTAAGATTCCAAGAAGATGAAATTAAGGAAAAGGAGGCTCAGGAGAAGCGGGAATCAACA
       D  E  C  L  V  R  F  Q  E  D  E  I  K  E  K  E  A  Q  E  K  R  E  S  T
1513  TGGAAGCGCTTGGAAGATGTGGCAGCCTCCAAGGCTGTTGTAAGTAATGAGGCTGTGCTTGTGAAGTTTGTC
       W  K  R  L  E  D  V  A  A  S  K  A  V  V  S  N  E  A  V  L  V  K  F  V
1585  TCTTCTGTTGCCATTGCTACCAACACAAATCTGCGAACAACTGTGGGTAGTTGA
       S  S  V  A  I  A  T  N  T  N  L  R  T  T  V  G  S  *
```

Figure S7. Nucleotide and deduced amino acid sequences of *CsSAND* from *C. sinensis*.

```
1       ATGGGGTCCGATTTGGACTCCTCAACGTCCTCCGACGATCCCATACACGAAAGCCCTAATTCTACTTCAATC
         M  G  S  D  L  D  S  S  T  S  S  D  D  P  I  H  E  S  P  N  S  T  S  I
73      GATCAGTCCCTCGACGCCATCGAAGATCAATTAGCCTCAATCGCATTGACCCAACCGAACGGCGTTGCAGCC
         D  Q  S  L  D  A  I  E  D  Q  L  A  S  I  A  L  T  Q  P  N  G  V  A  A
145     GACGATGAATCGTCCGAGCAAGAAGTTTTCAAGGACACCCCAAATGGGCTGCCTTTGGATGAGGACAGTAAT
         D  D  E  S  S  E  Q  E  V  F  K  D  T  P  N  G  L  P  L  D  E  D  S  N
217     CAGCAGGAGAGCAAGGGAGGTGAAATCGGAGAGGAGGGTTTTGGGAATTCAGTGCCTGAACCGTCTTCTGTG
         Q  Q  E  S  K  G  G  E  I  G  E  E  G  F  G  N  S  V  P  E  P  S  S  V
289     GAGTTTGAAGTTGTGGAGGAGGTTCGGGAGAGCTCGTCGAGTGGTGTTTTTGCGTGGAGGAGGACCAATTCG
         E  F  E  V  V  E  E  V  R  E  S  S  S  S  G  V  F  A  W  R  R  T  N  S
361     GAGCTCGAAGTTGAGCGGCAGGAGAGCCCAAGCAGTAGTGGGTATGCTGGTGAGAGAGGGAGTAGCAGTAGC
         E  L  E  V  E  R  Q  E  S  P  S  S  S  G  Y  A  G  E  R  G  S  S  S  S
433     ACCAGTGTGTCTGAGATTGAGGAGGTGGGTGAAGATCAAATTTGTGAAGTTGGGAAGGATGATTCTGTTGAT
         T  S  V  S  E  I  E  E  V  G  E  D  Q  I  C  E  V  G  K  D  D  S  V  D
505     GGGGGTTCGGATTGGGTTCCGGGGAAGCGGCATCTCGATGAAGATGATGCTTCTGTTTCATGGAGGAAAAGG
         G  G  S  D  W  V  P  G  K  R  H  L  D  E  D  D  A  S  V  S  W  R  K  R
577     AAGAAGCACTTCTTTATTTTGAGTAACTCTGGCAAACCAATATATTCCAGATACGGAGATGAACACAAGTTA
         K  K  H  F  F  I  L  S  N  S  G  K  P  I  Y  S  R  Y  G  D  E  H  K  L
649     GCGGGATTTTCGGCTACTCTGCAAGCAATCATTTCCTTCGTGGAGAATGGGGGCGATCGTGTGAAATTGGTA
         A  G  F  S  A  T  L  Q  A  I  I  S  F  V  E  N  G  G  D  R  V  K  L  V
721     AGGGGCAGGCAAACACCAGGTAATTTTTCTTGTGAAAGGACCAATCTACTTAGTTTGCATAAGCTGTACGGAG
         R   A  G  K  H  Q  V  I  F  L  V  K  G  P  I  Y  L  V  C  I  S  C  T  E
793     GAGCCTTATGAGTCATTAAGGGG*CAATTGGAGCTTGTTTATGGGCAGATGATAGTTATTCTTACAAAGTCT
         E  P  Y  E  S  L  R  *  Q  L  E  L  V  Y  G  Q  M  I  V  I  L  T  K  S
865     GTAAACAGATGTTTCGAGAAGAATTCAAAGTTTGATATGACACCTTTGCTTGGAGGAACAGATGTTGTCTTC
         V  N  R  C  F  E  K  N  S  K  F  D  M  T  P  L  L  G  G  T  D  V  V  F
937     TCTTCTCTCATCCATTCTTTCAGTTGGAACCCTGCCACTTTTCTTCATGCATACACGTGTCTTCCCGTTGCT
         S  S  L  I  H  S  F  S  W  N  P  A  T  F  L  H  A  Y  T  C  L  P  V  A
1009    TATGCGACAAGACAAGCTGCAGGTGCCATATTGCAAGATGTTGCTGATTCAGGTGTCCTGTTTGCAATATTA
         Y  A  T  R  Q  A  A  G  A  I  L  Q  D  V  A  D  S  G  V  L  F  A  I  L
1081    ATGTGCAAACACAAGGTTATCAGTCTTGTTGGTGCACAAAAAGCATCTCTTCATCCCGATGATATGCTCCTA
         M  C  K  H  K  V  I  S  L  V  G  A  Q  K  A  S  L  H  P  D  D  M  L  L
1153    CTTTCCAATTTTGTTATGTCATCGGAATCATTTAGGACATCTGAATCTTTCTCACCAATTTGCCTGCCAAGA
         L  S  N  F  V  M  S  S  E  S  F  R  T  S  E  S  F  S  P  I  C  L  P  R
1225    TATAATCCCATGGCATTTTTGTATGCTTATGTCCATTATCTTGATGTTGATACATACTTAATGTTGCTTACT
         Y  N  P  M  A  F  L  Y  A  Y  V  H  Y  L  D  V  D  T  Y  L  M  L  L  T
1297    ACCAGTTCAGATGCCTTTTTTCATCTTAAAGATTGCAGGATTCGTATCGAAATGGTCCTTTTGAAGTCAAAT
         T  S  S  D  A  F  F  H  L  K  D  C  R  I  R  I  E  M  V  L  L  K  S  N
1369    GTTCTCAGTGAAGTTCAGAAATCCATGCTAGATGGCGGCATGCGTGTTGAGGATTTGCCTGTTGATCCATCT
         V  L  S  E  V  Q  K  S  M  L  D  G  G  M  R  V  E  D  L  P  V  D  P  S
1441    CCTCGTTCTGGATATTCATCTGCTCATTTAGGTCAGCCCAGACTTGCAACAGATTCTCCTGAGAGATTCCGG
         P  R  S  G  Y  S  S  A  H  L  G  Q  P  R  L  A  T  D  S  P  E  R  F  R
1513    GAAGCATTTGTTGGTATTGGTGGTCCTGCTGGACTTTGGAATTTCATATATCGTAGTATTTACTTGGACCAA
         E  A  F  V  G  I  G  G  P  A  G  L  W  N  F  I  Y  R  S  I  Y  L  D  Q
1585    TATGTATCTTCTGAGTTCTCTTCGCCAATCAGTAGTTCGCGACAGCAGAAAAGATTGTATAGAGCTTACCAA
         Y  V  S  S  E  F  S  S  P  I  S  S  S  R  Q  Q  K  R  L  Y  R  A  Y  Q
1657    AAACTTTATGCCTCCATGCATGAGGAAGGAGTTGGACCCCACAAAACTCAGTTTAGAAGAGATGAGAACTAT
         K  L  Y  A  S  M  H  E  E  G  V  G  P  H  K  T  Q  F  R  R  D  E  N  Y
1729    GTTTTACTATGCTGGGTCACCCAGGACTTTGAACTCTATGCTGCATTTGATCCCCTGGCACACAAGGCTTTG
         V  L  L  C  W  V  T  Q  D  F  E  L  Y  A  A  F  D  P  L  A  H  K  A  L
1801    GCAATAAAGACTTGCAACCGGGTGTGTCAATGGGTGAAAGATGTGGAAAATGAGATCTTCTTGTTGGGAGCC
         A  I  K  T  C  N  R  V  C  Q  W  V  K  D  V  E  N  E  I  F  L  L  G  A
1873    AGCCCCTTTTCATGGTGA
         S  P  F  S  W  *
```

Figure S8. Nucleotide and deduced amino acid sequences of *CsTBP* from *C. sinensis*.

```
1     ATGGCGGATCAAGTGTTGGAAGGGAGCCAACCCGTGGATCTCTCCAAGCACCCGTCTGGCATCGTTCCTACT
       M  A  D  Q  V  L  E  G  S  Q  P  V  D  L  S  K  H  P  S  G  I  V  P  T
73    CTTCAGAATATTGTGTCAACAGTGAACCTCGATTGCAAGTTAGATCTCAAAGCCATTGCTTTGCAAGCTCGT
       L  Q  N  I  V  S  T  V  N  L  D  C  K  L  D  L  K  A  I  A  L  Q  A  R
145   AATGCCGAATACAATCCCAAGCGTTTTGCTGCTGTTATTATGAGGATAAGGGATCCAAAGACGACAGCTTTG
       N  A  E  Y  N  P  K  R  F  A  A  V  I  M  R  I  R  D  P  K  T  T  A  L
217   ATTTTTGCCTCTGGGAAGATGGTTTGCACTGGTGCAAAGAGCGAACAACTTTCAAAACTGGCAGCAAGAAAG
       I  F  A  S  G  K  M  V  C  T  G  A  K  S  E  Q  L  S  K  L  A  A  R  K
289   TATGCACGAATTATTCAAAAGCTTGGGTTTCCTGCCAAATTCAAGGATTTCAAGATTCAGAACATAGTTGGT
       Y  A  R  I  I  Q  K  L  G  F  P  A  K  F  K  D  F  K  I  Q  N  I  V  G
361   TCCTGTGACGTAAAATTTCCCATCAGACTTGAAGGTCTTGCATATTCCCATGGTGCCTTTTCAAGTTATGAA
       S  C  D  V  K  F  P  I  R  L  E  G  L  A  Y  S  H  G  A  F  S  S  Y  E
433   CCAGAACTATTTCCAGGCTTAATATATCGTATGAAGCAACCAAAGATTGTGCTACTCATCTTTGTTTCTGGA
       P  E  L  F  P  G  L  I  Y  R  M  K  Q  P  K  I  V  L  L  I  F  V  S  G
505   AAGATTGTCCTTACGGGAGCCAAGGTGAGGGATGAGACATATACAGCCTTCGAGAACATATACCCTGTCCTT
       K  I  V  L  T  G  A  K  V  R  D  E  T  Y  T  A  F  E  N  I  Y  P  V  L
577   ACTGAGTTCAGGAAGAATCAACAATGGTAG
       T  E  F  R  K  N  Q  Q  W  *
```

Figure S9. Nucleotide and deduced amino acid sequences of *CsTIP41* from *C. sinensis*.

```
1     ATGGAGTTGGAAGTGGACGAGACCGAGCTCAAAGCCGCCGGAGCTCAGCTTCTTCCGGAAGGACGCGTTGGA
       M  E  L  E  V  D  E  T  E  L  K  A  A  G  A  Q  L  L  P  E  G  R  V  G
73    CTCTTCATACATGGTTGGGAGATCGAGTCTCGCAAGCGTCCCATTCTCAACTCTCTCCACCTCCAACAGTGG
       L  F  I  H  G  W  E  I  E  S  R  K  R  P  I  L  N  S  L  H  L  Q  Q  W
145   GAGCATAAGCTTCAAACATCCCACTTTCCAGAGATGGTTTTTGGGGACAGTTCTTTGGTTCTTAAACATGTC
       E  H  K  L  Q  T  S  H  F  P  E  M  V  F  G  D  S  S  L  V  L  K  H  V
217   AGTAGTGGCATTAAGATTCATTTTAATACATTTGATGCTCTAACTGCCTGGAAGCAGGAAGCATTGCCACCA
       S  S  G  I  K  I  H  F  N  T  F  D  A  L  T  A  W  K  Q  E  A  L  P  P
289   GTTGAAGTTCCTGCAGCTGCAAAATGGAAATTCAGAAGCCAACCCTTCCAGCAATTGGATTATGACTATACC
       V  E  V  P  A  A  A  K  W  K  F  R  S  Q  P  F  Q  Q  L  D  Y  D  Y  T
361   TTTACAACACCATATTGTGGAAGTCAAACGATTGAGATAAATGAAGAGCTTGCGAGAGGAGCAACCTCTGAA
       F  T  T  P  Y  C  G  S  Q  T  I  E  I  N  E  E  L  A  R  G  A  T  S  E
433   GATAACAACTGTGGTCTTTATTGGGAGGACTGCAAAGAGCAAATTGATTTGGCTGCACTGGCATCGAAAGAG
       D  N  N  C  G  L  Y  W  E  D  C  K  E  Q  I  D  L  A  A  L  A  S  K  E
505   CCCATTCTCTTCTATGATGAGGTAATCTTGTATGAAGATGAATTGGCGGATAGTGGTGTTTCACTTTTAACT
       P  I  L  F  Y  D  E  V  I  L  Y  E  D  E  L  A  D  S  G  V  S  L  L  T
577   GTAAAAGTGAGGGTAATGCCAAACTCTTGGTTTCTTCTCTTGCGTTTTTGGCTTAGAGTTGATGGGGTGCTT
       V  K  V  R  V  M  P  N  S  W  F  L  L  L  R  F  W  L  R  V  D  G  V  L
649   ATGAGATTGAGGGACACACGTATGCATTGTGTTTTTGCAAATAGTGCAACCCCCATTATTCTTCGAGAAAGC
       M  R  L  R  D  T  R  M  H  C  V  F  A  N  S  A  T  P  I  I  L  R  E  S
721   TGTTGGAGGGAAGCCACATTTAAAGCTTTGTCTGCAAAAGGATACCCATCTGATTCTGCGGCATATAATGAT
       C  W  R  E  A  T  F  K  A  L  S  A  K  G  Y  P  S  D  S  A  A  Y  N  D
793   CCAAGCATCATCAGCCAGAGGCTTCCTATCATCATGCATAAGTCCCAAAAGCTTAAAATCCCTGGTAACCTA
       P  S  I  I  S  Q  R  L  P  I  I  M  H  K  S  Q  K  L  K  I  P  G  N  L
865   TAA
       *
```

Figure S10. Nucleotide and deduced amino acid sequences of *CsTUB* from *C. sinensis*.

```
1       ATGAGAGAGATTCTTCACATTCAAGGTGGACAGTGCGGAAATCAGATCGGATCAAAATTTTGGGAAGTTGTT
         M  R  E  I  L  H  I  Q  G  G  Q  C  G  N  Q  I  G  S  K  F  W  E  V  V
73      TGTGATGAACATGGCATAGATCCTACTGGAAGATATGTTGGAACTTCAGATTTGCAGCTCGAACGTGTCAAT
         C  D  E  H  G  I  D  P  T  G  R  Y  V  G  T  S  D  L  Q  L  E  R  V  N
145     GTCTACTACAATGAGGCTTCTTGTGGGAGGTTTGTTCCTCGTGCTGTGCTCATGGATCTTGAGCCGGGCACC
         V  Y  Y  N  E  A  S  C  G  R  F  V  P  R  A  V  L  M  D  L  E  P  G  T
217     ATGGACAGTGTTCGCACTGGTCCGTATGGCCAGATCTTCCGCCCTGATAACTTTGTTTTCGGTCAGTCTGGT
         M  D  S  V  R  T  G  P  Y  G  Q  I  F  R  P  D  N  F  V  F  G  Q  S  G
289     GCTGGAAATAACTGGGCCAAAGGACATTACACTGAGGGTGCAGAACTTATTGATTCAGTTCTTGATGTTGTG
         A  G  N  N  W  A  K  G  H  Y  T  E  G  A  E  L  I  D  S  V  L  D  V  V
361     AGGAAGGAGGCCGAGAACTGTGACTGTCTTCAAGGTTTTCAAGTCTGCCATTCCCTGGGTGGAGGAACAGGT
         R  K  E  A  E  N  C  D  C  L  Q  G  F  Q  V  C  H  S  L  G  G  G  T  G
433     TCTGGGATGGGTACCTTGCTAATTTCAAAAATTAGGGAGGAGTACCCTGACAGGATGATGCTCACATTCTCT
         S  G  M  G  T  L  L  I  S  K  I  R  E  E  Y  P  D  R  M  M  L  T  F  S
505     GTGTTCCCATCGCCAAAGGTTTCAGATACTGTTGTTGAGCCATACAATGCTACCCTTTCTGTCCACCAGCTT
         V  F  P  S  P  K  V  S  D  T  V  V  E  P  Y  N  A  T  L  S  V  H  Q  L
577     GTTGAGAATGCAGATGAGTGCATGGTGCTTGATAATGAAGCTCTATATGATATCTGTTTCAGGACTCTTAAG
         V  E  N  A  D  E  C  M  V  L  D  N  E  A  L  Y  D  I  C  F  R  T  L  K
649     CTTACCACTCCTAGCTTTGGCGATCTGAACCACTTGATATCTGCAACCATGAGTGGTGTCACTTGCTGCCTT
         L  T  T  P  S  F  G  D  L  N  H  L  I  S  A  T  M  S  G  V  T  C  C  L
721     CGATTCCCTGGTCAACTCAATTCTGATCTCCGAAAGCTTGCTGTAAACCTTATCCCTTTTCCCCGTCTACAC
         R  F  P  G  Q  L  N  S  D  L  R  K  L  A  V  N  L  I  P  F  P  R  L  H
793     TTCTTCATGGTGGGTTTTGCTCCGCTGACTTCACGTGGGTCCCAGCAATACCGAGCCCTTACTGTCCCTGAA
         F  F  M  V  G  F  A  P  L  T  S  R  G  S  Q  Q  Y  R  A  L  T  V  P  E
865     CTGACCCAACAAATGTGGGATGCAAAGAACATGATGTGTGCTGCTGACCCACGACATGGCCGCTACCTCACT
         L  T  Q  Q  M  W  D  A  K  N  M  M  C  A  A  D  P  R  H  G  R  Y  L  T
937     GCTTCAGCCATGTTCAGGGGTAAAATGAGCACCAAGGAAGTGGATGAACAAATGATCAACGTTCAAAACAAG
         A  S  A  M  F  R  G  K  M  S  T  K  E  V  D  E  Q  M  I  N  V  Q  N  K
1009    AACTCTTCTTACTTTGTGGAGTGGATCCCTAACAATGTGAAATCGAGCGTCTGTGACATTCCACCTAGGGGA
         N  S  S  Y  F  V  E  W  I  P  N  N  V  K  S  S  V  C  D  I  P  P  R  G
1081    CTTTCCATGGCATCAACCTTTATTGGGAATTCAACCTCCATTCAGGAAATGTTCAGGAGAGTGAGTGAGCAG
         L  S  M  A  S  T  F  I  G  N  S  T  S  I  Q  E  M  F  R  R  V  S  E  Q
1153    TTCACTGCTATGTTCAGGAGGAAGGCTTTCTTGCATTGGTATACTGGCGAAGGTATGGATGAAATGGAGTTC
         F  T  A  M  F  R  R  K  A  F  L  H  W  Y  T  G  E  G  M  D  E  M  E  F
1225    ACAGAAGCCGAGAGCAACATGAATGATCTAGTGTCGGAGTACCAGCAGTACCAGGATGCTACTGCTGATGAG
         T  E  A  E  S  N  M  N  D  L  V  S  E  Y  Q  Q  Y  Q  D  A  T  A  D  E
1297    GAGGGCGAATATGATGATGAGGAGGAAGAAGGTATGGAGGACATGTGA
         E  G  E  Y  D  D  E  E  E  E  G  M  E  D  M  *
```

Figure S11. Amplification of qRT-PCR specific primers.

Amplified fragments of nine candidate reference genes in *C. sinensis* were separated by 1.2% agarose gel electrophoresis. M: marker 2000.

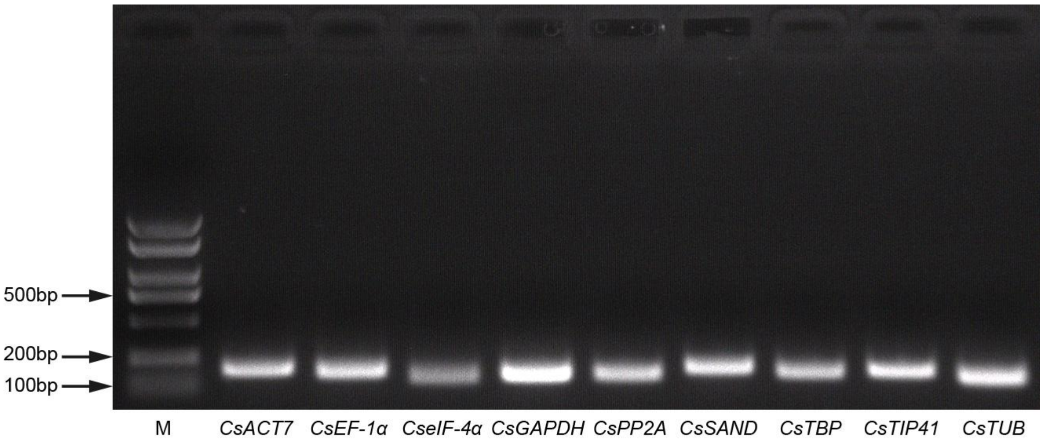

Figure S12. Standard curves of each candidate genes.

Standard curves of *CsACT7*, *CsEF-1α*, *CseIF-4α,* *CsGAPDH*, *CsPP2A*, *CsSAND*, *CsTBP*, *CsTIP41*, and *CsTUB*. The linear correlation (R^2) and PCR efficiencies (% E = $(10^{[-1/\text{slope}]} - 1) \times 100\%$) were calculated from the standard curve.

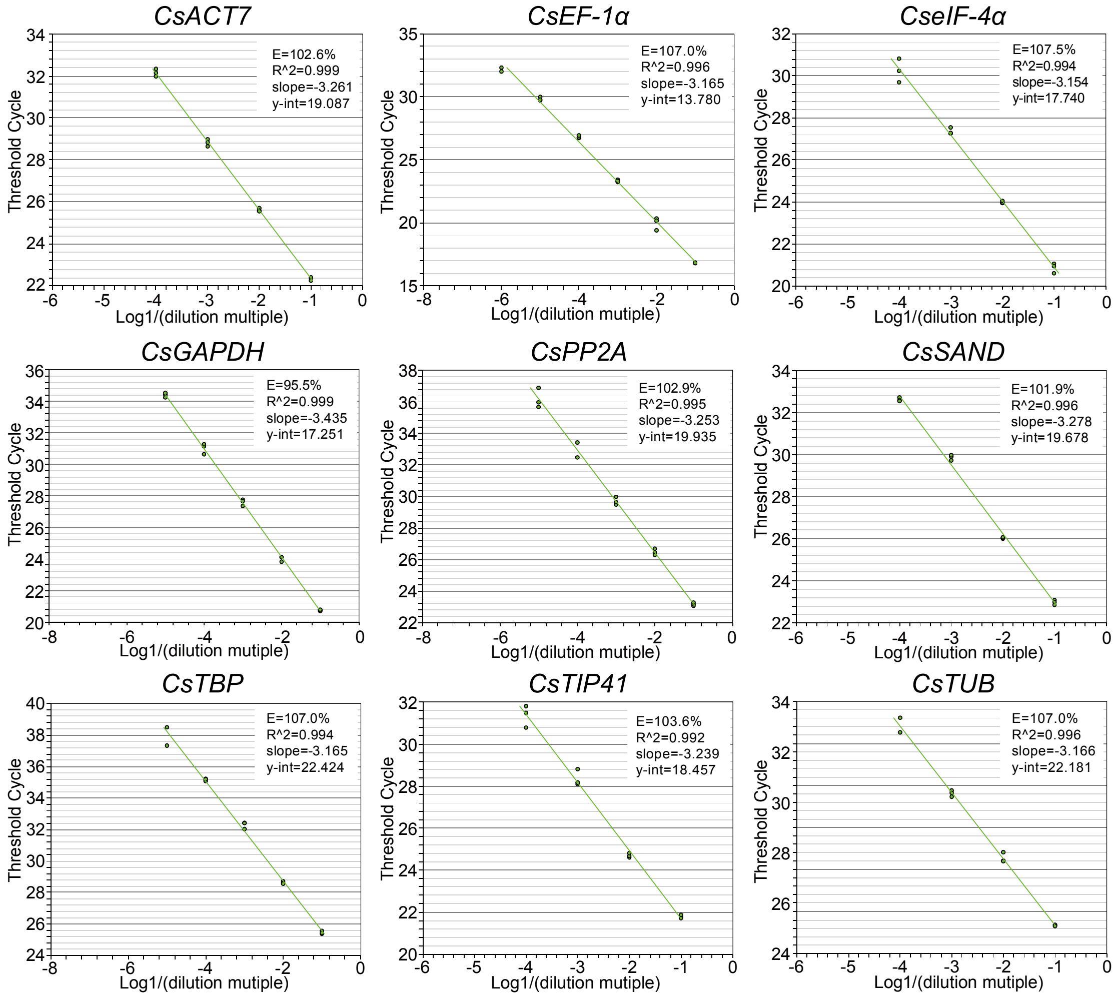

Table S1. Primer sequences for cloning of nine reference genes from *C. sinensis*

Gene symbol	Primer sequence (5'–3') forward/reverse	Amplicon length (bp)	*Arabidopsis* homolog gene	Identity (%)
CsACT7	ATGGCTGATGCTGAGGATATTCAGC<br>TCAAAAGCACTTCCTGTGGACAATG	1134	AT5G09810	96.82
CsEF-1α	ATGGGTAAGGAAAAAGTTCACATTA<br>TCATTTCTTCTTCAGAGCAGCCTT	1344	AT5G60390	95.77
CseIF-4α	ATGGCTGGCTTGGCACCTGAG<br>TCAAAGGAGATCAGCAACATTTGCT	1241	AT3G13920	92.74
CsGAPDH	ATGATAACTGAAAAAACAAATGTAA<br>TTAAGCTTCATAAACCTTGCACT	1368	AT1G42970	83.99
CsPP2A	ATGATTAGGCAGATTCTGGGTAAGC<br>TCAACTACCCACAGTTGTTCGCAG	1638	AT3G21650	73.32
CsSAND	ATGGGGTCCGATTTGGACTCCT<br>TCACCATGAAAAGGGGCTGGCT	1890	AT2G28390	64.09
CsTBP	ATGGCGGATCAAGTGTTGGAA<br>CTACCATTGTTGATTCTTCCTGAAC	606	AT1G55520	93.03
CsTIP41	ATGGAGTTGGAAGTGGACGAGA<br>TTATAGGTTACCAGGGATTTTAAGC	867	AT4G34270	67.24
CsTUB	ATGAGAGAGATTCTTCACATTCAAG<br>TCACATGTCCTCCATACCTTCTTCC	1344	AT5G12250	96.66

Table S2. Raw Cq values of nine reference genes in *C. sinensis*.

Plant material samples from different development leaves and hormone treatments.

	CsACT7	*CsEF-1α*	*CseIF-4α*	*CsGAPDH*	*CsPP2A*	*CsSAND*	*CsTBP*	*CsTIP41*	*CsTUB*
First-leaf-1	23.00	20.08	24.78	23.53	26.76	25.56	25.54	25.08	22.62
First-leaf-2	23.24	20.39	24.59	23.47	26.66	25.67	25.42	24.93	22.51
First-leaf-3	22.78	20.09	24.36	23.19	26.49	25.04	25.21	24.78	22.30
First-leaf-4	22.51	19.38	23.75	22.43	26.37	25.51	25.13	24.67	21.74
First-leaf-5	22.66	19.27	23.75	22.48	26.30	25.21	25.00	24.56	21.75
First-leaf-6	22.55	19.24	23.82	22.66	26.33	25.13	24.98	24.59	21.85
First-leaf-6	23.29	19.82	24.53	23.04	26.91	25.93	25.64	25.39	22.67
First-leaf-8	23.69	20.20	24.72	23.40	27.28	26.21	25.82	25.68	22.89
First-leaf-9	23.70	20.21	24.55	23.36	27.02	26.17	25.74	25.50	22.98
Second-leaf-1	22.37	19.70	24.41	22.00	26.07	24.85	25.08	24.55	22.22
Second-leaf-2	22.43	19.78	24.07	22.08	26.21	24.99	25.14	24.74	22.35
Second-leaf-3	22.65	19.75	24.18	22.18	26.27	25.00	25.12	24.69	22.39
Second-leaf-4	23.17	20.13	24.55	21.84	26.78	25.85	25.85	25.41	22.82
Second-leaf-5	22.85	19.80	24.37	21.68	26.70	25.83	25.63	25.11	22.65
Second-leaf-6	22.87	19.86	24.23	21.51	26.58	25.78	25.47	25.13	22.47
Second-leaf-7	22.46	19.35	23.94	21.84	26.18	24.93	24.96	24.55	22.10
Second-leaf-8	22.60	19.47	23.95	21.88	26.21	25.05	25.11	24.60	22.06
Second-leaf-9	22.65	19.68	24.06	22.07	26.35	25.08	25.08	24.67	22.26
Third-leaf-1	22.71	20.40	24.68	21.80	26.40	25.36	25.49	25.05	22.98
Third-leaf-2	22.68	20.28	24.48	21.73	26.29	25.06	25.31	24.88	22.76
Third-leaf-3	22.66	20.11	24.44	21.70	26.34	25.08	25.28	24.89	22.75
Third-leaf-4	22.84	20.33	24.71	21.43	26.75	25.61	25.56	25.14	22.83
Third-leaf-5	22.61	20.29	24.64	21.39	26.60	25.62	25.55	25.19	22.88
Third-leaf-6	22.73	20.36	24.65	21.34	26.56	25.57	25.53	25.28	22.85
Third-leaf-7	22.08	19.98	24.04	21.17	25.82	24.71	24.95	24.47	22.37
Third-leaf-8	22.29	20.05	23.92	21.12	25.67	24.73	25.08	24.05	22.46
Third-leaf-9	22.20	19.94	24.09	21.23	25.85	24.72	24.95	24.47	22.52
Fourth-leaf-1	22.34	20.33	24.46	21.96	26.52	25.53	25.54	24.77	23.09
Fourth-leaf-2	22.00	19.98	24.08	21.60	26.09	25.11	25.21	24.60	22.73
Fourth-leaf-3	21.86	19.80	24.01	21.39	25.99	24.94	25.00	24.40	22.56
Fourth-leaf-4	21.70	20.02	24.16	20.73	26.30	25.37	25.32	24.70	22.77
Fourth-leaf-5	21.50	19.86	24.17	20.68	26.33	25.38	25.17	24.38	22.68
Fourth-leaf-6	21.74	20.12	24.25	20.69	26.32	25.65	25.36	24.68	22.77
Fourth-leaf-7	22.24	20.26	24.43	21.39	26.38	25.92	25.54	25.09	23.05
Fourth-leaf-8	22.20	20.10	24.52	21.38	26.37	25.79	25.55	25.08	23.12
Fourth-leaf-9	22.46	20.51	24.72	21.71	26.59	26.00	25.68	25.31	23.33
Older-leaf-1	23.70	22.64	26.69	21.95	29.73	28.26	28.10	26.64	29.53
Older-leaf-2	23.80	22.50	26.53	21.85	29.61	28.05	28.12	26.39	29.09

Older-leaf-3	23.54	22.24	26.52	21.78	29.19	27.85	27.92	26.36	29.18
Older-leaf-4	22.24	21.33	25.55	20.97	27.80	26.67	26.60	25.43	27.45
Older-leaf-5	22.11	21.01	25.25	20.80	27.99	26.42	26.42	25.05	27.20
Older-leaf-6	22.65	21.37	25.85	20.75	27.99	26.71	26.68	25.21	27.61
Older-leaf-7	23.11	21.82	26.14	21.34	29.22	28.07	27.38	26.11	28.69
Older-leaf-8	23.11	21.87	26.02	21.34	29.16	27.98	27.63	26.07	28.73
Older-leaf-9	23.02	21.73	26.17	21.43	29.49	28.17	27.62	26.28	28.86
ABA-1	23.43	20.57	24.95	21.81	27.05	26.40	25.81	25.79	23.24
ABA-2	23.45	20.67	24.84	21.69	26.93	26.31	25.74	25.71	23.14
ABA-3	23.34	20.39	24.74	21.65	26.88	26.24	25.60	25.58	23.06
ABA-4	22.82	20.18	24.60	21.67	25.90	25.22	25.00	24.83	22.19
ABA-5	22.74	20.06	24.44	21.38	25.80	25.24	24.87	24.49	22.18
ABA-6	22.75	19.97	24.49	21.64	25.85	25.37	24.73	24.72	22.23
ABA-7	22.39	19.82	23.89	21.07	25.66	24.71	24.51	24.52	21.92
ABA-8	22.27	19.78	23.94	20.91	25.68	24.74	24.43	24.35	21.80
ABA-9	22.44	19.53	23.46	21.00	25.46	24.46	24.41	24.38	21.91
GA-1	23.04	20.36	24.57	21.03	26.48	26.41	25.27	25.16	22.16
GA-2	23.14	20.16	24.27	21.03	26.61	26.13	25.30	25.28	22.30
GA-3	23.07	20.35	24.73	21.23	26.63	26.33	25.72	25.39	22.22
GA-4	22.99	20.29	24.72	21.25	26.18	25.73	25.15	25.13	22.68
GA-5	23.26	20.69	24.86	21.56	26.31	26.04	25.47	25.43	22.75
GA-6	23.22	20.31	24.66	21.30	26.22	25.93	25.39	25.29	22.69
GA-7	23.81	21.55	25.60	22.12	27.74	27.47	26.48	25.46	24.12
GA-8	24.40	21.95	26.22	22.67	28.37	27.84	26.72	25.86	24.69
GA-9	23.99	21.80	25.99	22.47	28.11	27.65	26.83	25.75	24.62
IAA-1	24.67	20.22	24.21	21.63	26.66	25.87	25.85	25.89	23.14
IAA-2	24.66	20.18	24.27	21.51	26.62	25.76	25.83	25.76	23.00
IAA-3	24.77	20.16	24.56	21.69	26.50	25.45	25.50	25.70	22.97
IAA-4	23.45	20.26	24.56	21.37	26.38	25.85	25.43	25.35	22.53
IAA-5	23.12	20.10	24.66	21.32	26.48	25.69	25.25	25.36	22.52
IAA-6	22.86	19.97	24.43	21.00	26.35	25.70	25.73	25.23	22.56
IAA-7	23.06	20.11	24.71	21.08	27.15	26.29	25.81	25.54	22.87
IAA-8	23.17	20.15	24.63	20.82	26.96	26.25	25.82	25.65	22.99
IAA-9	23.25	20.31	24.69	20.95	26.91	26.50	25.85	25.80	23.10
MJ-1	23.12	20.78	24.96	21.47	27.38	26.72	25.98	25.87	23.30
MJ-2	22.89	20.24	24.50	20.99	26.82	26.17	25.70	25.50	22.92
MJ-3	22.87	20.34	24.59	20.89	26.91	26.32	25.72	25.48	22.97
MJ-4	23.14	20.58	24.64	21.30	26.72	26.10	25.63	25.20	23.61
MJ-5	23.06	20.66	24.82	21.14	26.67	26.10	25.52	25.20	23.53
MJ-6	23.03	20.38	24.52	20.96	26.50	26.07	25.41	25.19	23.26
MJ-7	22.35	19.67	24.08	21.78	26.05	25.09	24.82	24.61	23.09
MJ-8	22.32	19.68	24.11	21.78	25.92	25.28	24.93	24.57	23.30
MJ-9	22.10	19.52	24.56	21.65	25.78	25.01	24.79	24.53	22.87
SA-1	23.21	20.02	24.66	21.05	27.02	26.31	25.90	25.33	23.53

SA-2	23.65	20.47	24.79	21.26	27.19	26.59	26.23	25.43	23.67
SA-3	23.71	20.44	25.07	21.41	27.35	26.70	26.02	25.68	23.70
SA-4	23.30	20.42	24.95	20.48	26.87	26.26	25.58	25.48	23.55
SA-5	23.32	20.52	24.98	20.52	26.96	26.40	25.73	25.68	23.67
SA-6	23.51	20.79	25.14	20.48	27.21	26.38	25.96	25.78	23.80
SA-7	22.65	20.00	24.33	20.49	26.53	25.99	25.25	24.27	22.94
SA-8	22.47	20.78	25.00	20.33	27.27	26.72	25.86	25.08	23.97
SA-9	22.51	20.01	24.42	20.54	26.68	26.11	25.42	24.48	23.08

Table S3. Data statistics of Cq values of candidate reference genes.

	N Total	Median	Ar Mean	Std Dev	Minimum	Maximum
CsACT7	72	22.87	22.92	1.41	21.5	24.77
CsEF-1α	72	20.21	20.34	1.39	19.24	22.64
CseIF-4α	72	24.56	24.66	1.37	23.46	26.69
CsGAPDH	72	21.42	21.54	1.43	20.33	23.53
CsPP2A	72	26.59	26.78	1.54	25.46	29.73
CsSAND	72	25.85	25.93	1.59	24.46	28.26
CsTBP	72	25.53	25.64	1.43	24.41	28.12
CsTIP41	72	25.19	25.17	1.36	24.05	26.64
CsTUB	72	22.88	23.4	2.21	21.74	29.53
